# Comprehensive and systematic characterization of multi-functionalized cisplatin nano-conjugate: from the chemistry and proteomic biocompatibility to the animal model

**DOI:** 10.1186/s12951-022-01546-y

**Published:** 2022-07-20

**Authors:** Ángela-Patricia Hernández, Ania Micaelo, Rafael Piñol, Marina L. García-Vaquero, José J. Aramayona, Julio J. Criado, Emilio Rodriguez, José Ignacio Sánchez-Gallego, Alicia Landeira-Viñuela, Pablo Juanes-Velasco, Paula Díez, Rafael Góngora, Ricardo Jara-Acevedo, Alberto Orfao, Javier Miana-Mena, María Jesús Muñoz, Sergio Villanueva, Ángel Millán, Manuel Fuentes

**Affiliations:** 1 Department of Medicine and General Cytometry Service-Nucleus, CIBERONC CB16/12/00400, Cancer Research Centre, (IBMCC/CSIC/USAL/IBSAL), University of Salamanca-CSIC, IBSAL, Campus Miguel de Unamuno s/n, 37007 Salamanca, Spain; 2 Department of Pharmaceutical Sciences. Organic Chemistry Section. Faculty of Pharmacy, University of Salamanca, Campus Miguel de Unamuno s/n, 37007 Salamanca, Spain; 3 INMA, Institute of Nanoscience and Materials of Aragon, CSIC-University of Zaragoza, 50018 Saragossa, Spain; 4 Department of Pharmacology and Physiology, University of Zaragoza, Zaragoza, Spain; 5 Department of Inorganic Chemistry, Faculty of Chemical Sciences, Plaza de los Caídos S/N, 37008 Salamanca, Spain; 6 ImmunoStep, SL, Edificio Centro de Investigación del Cáncer, University of Salamanca, Avda. Coimbra s/n, Campus Miguel de Unamuno, 37007 Salamanca, Spain; 7 Proteomics Unit, Cancer Research Centre (IBMCC/CSIC/USAL/IBSAL), 37007 Salamanca, Spain

**Keywords:** Nanoparticles, Cisplatin, Iron oxide nanoparticles, Protein corona, Drug delivery, Biocompatibility, Proteomics profiling, Cytotoxicity, Cell signaling, Animal model

## Abstract

**Background:**

Nowadays, nanoparticles (NPs) have evolved as multifunctional systems combining different custom anchorages which opens a wide range of applications in biomedical research. Thus, their pharmacological involvements require more comprehensive analysis and novel nanodrugs should be characterized by both chemically and biological point of view. Within the wide variety of biocompatible nanosystems, iron oxide nanoparticles (IONPs) present mostly of the required features which make them suitable for multifunctional NPs with many biopharmaceutical applications.

**Results:**

Cisplatin-IONPs and different functionalization stages have been broadly evaluated. The potential application of these nanodrugs in onco-therapies has been assessed by studying in vitro biocompatibility (interactions with environment) by proteomics characterization the determination of protein corona in different proximal fluids (human plasma, rabbit plasma and fetal bovine serum),. Moreover, protein labeling and LC–MS/MS analysis provided more than 4000 proteins de novo synthetized as consequence of the nanodrugs presence defending cell signaling in different tumor cell types (data available via ProteomeXchanges with identified PXD026615). Further in vivo studies have provided a more integrative view of the biopharmaceutical perspectives of IONPs.

**Conclusions:**

Pharmacological proteomic profile different behavior between species and different affinity of protein coating layers (soft and hard corona). Also, intracellular signaling exposed differences between tumor cell lines studied. First approaches in animal model reveal the potential of theses NPs as drug delivery vehicles and confirm cisplatin compounds as strengthened antitumoral agents.

**Supplementary Information:**

The online version contains supplementary material available at 10.1186/s12951-022-01546-y.

## Background

Nanoparticles (NPs) as drug delivery systems has been reported for the last decades [1]. However, only recently it has become a more widespread research field, as more advantages and new techniques are discovered and easily implemented in the biomedical field [2]. Since the first approval clinical use, NPs have become an essential axis for the multiple functional combination in a single construction that allows its versatile and efficient use. Nowadays, organic, inorganic and hybrid nanostructures can be design to optimizer physical and chemical characteristics (ie. size, form, surface charge, subtly modulated functionalization) for several purposes from early diagnosis to therapies (targeted administration of medicines, genetic and cellular therapy, molecular imagine, etc.). This customization allows to generate biocompatible and stable nanomaterials that can cross biological barriers to specifically reach the drug target [3–6].

Within the wide variety of biocompatible nanosystems, iron oxide nanoparticles (IONPs) present mostly of the required features which make them suitable for multifunctional NPs with a wide range of biopharmaceutical applications. These IONPs, with the proper surface polymer coating and chemical moieties, can be endorsed with blood biocompatibility [7] and excellent in vivo biodistribution [8]; while their magnetic properties can be intact, which are useful in diagnostics and therapy ( ie. as magnetic resonance image contrast agents, hyperthermia) [9, 10]. In addition, these nanoconstructs could be functionalized with a huge variety of chemical moieties and /or molecules such as fluorophores for optical imaging [11], radionuclides for single-photon emission computed tomography (SPECT) imaging [8], and even molecular thermosensors for non-contact temperature measurements [12], and chemical linkers for further biomolecule multi-functionalization (such as peptides, antibodies, oligonucleotides…) [13, 14].

Previous studies combining NPs and platinum derivates conjugated with bile acids (BilPt) have described interesting properties about the antitumoral activity for the resulting nano-conjugate (platinum precursor bound to NPs). Moreover, several relevant characteristics of IONPs have been observed such as vesicular transport and release capacity of platinum derivatives to the targeted cells [15, 16].

Despite the great advances in onco-immunotherapy by these nanodrugs conjugates (as by combination with immune-checkpoint inhibitors), only a small fraction of the proteome related to these nanodrugs is understood at biochemical level on tumoral cell of interest [16–18]. Systems biology and proteomics strive to create a detailed predictive model of molecular and intracellular pathways (based upon the dynamic networks), which provides clues into the consequence and/or effect of the nanodrug on the tumoral cells. However, collecting systematic biochemical data -at scale- about protein behavior has been dauting for these novel nanodrugs conjugates. In addition, the overwhelming size and complexity of human proteome requires very high-throughput techniques for deep and rapid analysis to determine NP interactions with environment. In this sense, protein corona has become as an essential component of NP interactions to understand the biocompatibility and to predict pharmacokinetics in blood [17]. It has been shown that the translation of NPs to the clinic has failed in many cases because of the difference in protein-corona interactions between in vitro and in vivo studies [17, 19]. Previous reports have used proteomic techniques for the determination of the protein corona and have also determined the discrimination between the layers formed around the NP and the protein corona [20, 21].

In this study, it is performed a systematically characterization by high-content quantitative proteomics strategies in order to decipher protein corona complexity in different biological fluids as wells as cytotoxic intracellular pathways to develop an integrated understanding of the behavior of a nanoconjugate as an entity, using IONPs as a multifunctional model. The potential of this IONPs, as effective anti-tumoral drug delivery system- has been also in vivo evaluated in an animal model displaying an outstanding correlation with this exhaustive proteomic characterization.

## Results

**Fig. 1:**
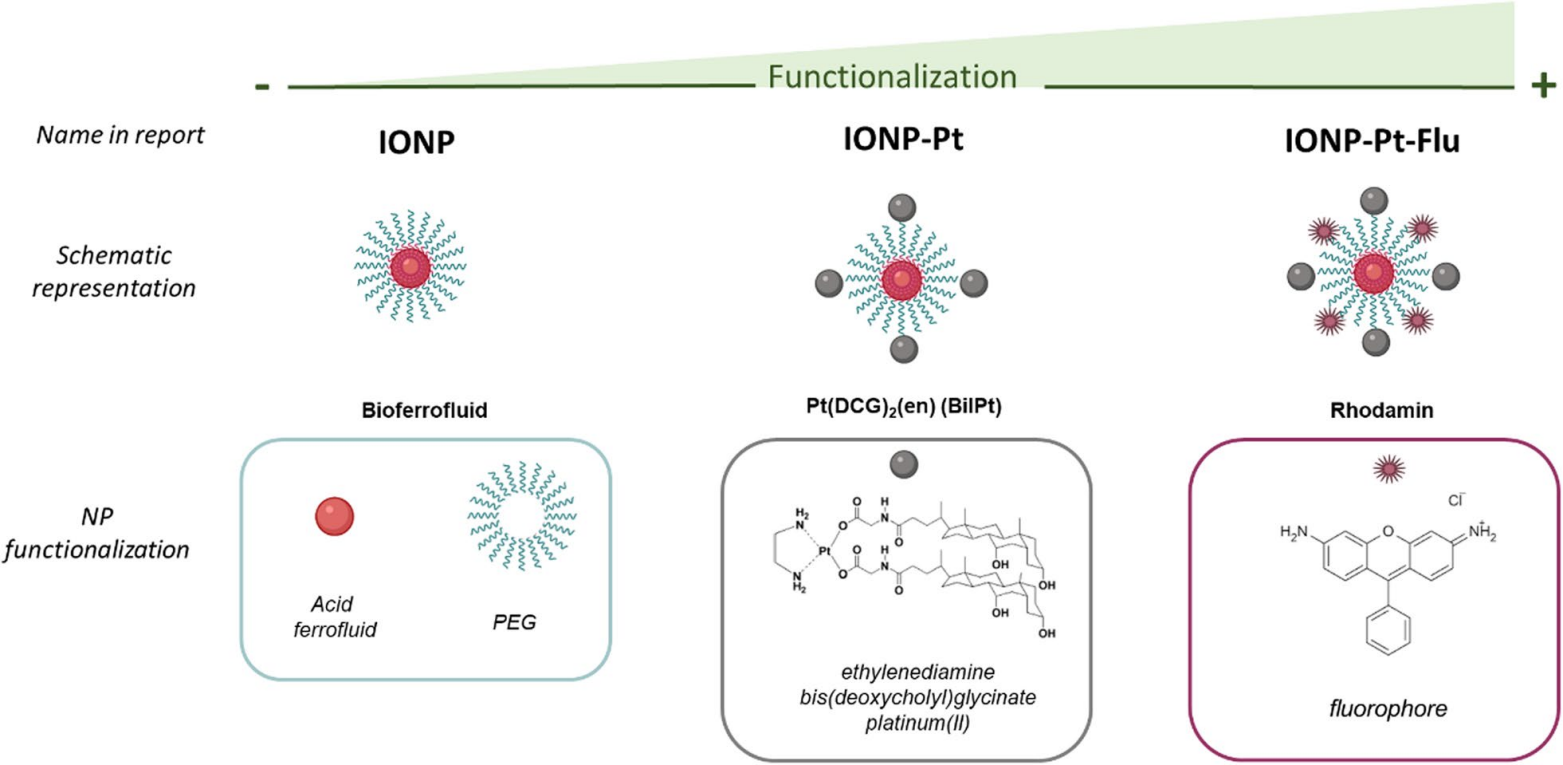
Schematic representation of NPs and their corresponding multifunctionalities tested in this report based on previous reports [16]

Based on previous results of IONPs activity [16], in this study, it has been explored systematic and exhaustive proteomics characterization of different nano-constructions in order to identify differences and similarities between them, useful in further characterizations of biological activity or possible biomedical application. In Fig. 1, it is schematically depicted the IONPs studied as model in this report, displaying the associated nomenclature to facilitate the follow-up and outcomes observed from the protein corona characterization and other screening approaches.

### Biological interactions and biocompatibility of nanodrugs conjugates based on multifunctional copolymer coated iron oxide NPs and platinum derivatives

Firstly, it is required the evaluation of the interaction with biomolecules contained on the biological fluids, which inherently bound to the surface of the nanodrug conjugate, known as protein corona. During last decade, several methodological strategies have been proposed to determine protein corona and further determination of differences in the intrinsic interaction between the NPs and the biological milieus, which can ultimately define their pharmacological activity [17, 22]. Among others described methodologies, high-throughput techniques are required because the wide molecular variety and multi-functionalities of the NPs.

**Fig. 2:**
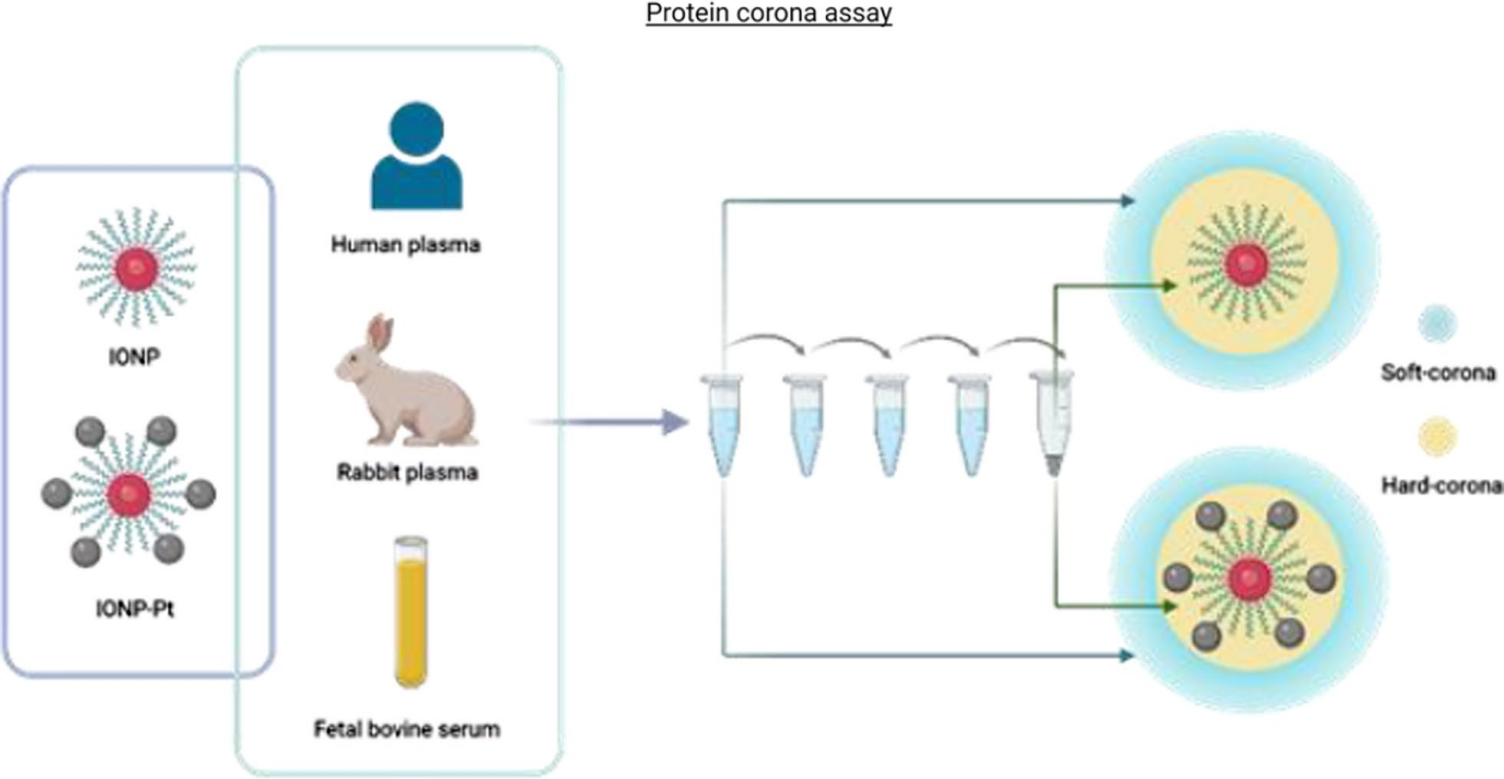
Schematic workflow of protein corona determination carried out to identify differences between soft- and hard-corona in different plasma samples (human, rabbit and FBS)

As previously mentioned, the protein corona (interaction between NPs surface and the physiological environment) is playing a critical role in the pharmacokinetics and pharmacodynamics of any novel nanodrugs conjugates, as it is directly related with functional process (such as blood absorption and distribution, to cellular endocytosis and pharmacological response, among others…) and the different wide dynamic range of biomolecules on biological proximal fluid, which are in continuous dynamic equilibrium between soluble and adsorbed biomolecules [17, 18]. Consequently, several coating layers constitute the protein corona depending on the NPs surface and molecules diversity. Thus, NPs are coated with a dynamic layer conformed by the most abundant proteins that is named as “soft-corona”. Gradually, some of these proteins are replaced by low abundant proteins but higher affinity for the NP, resulting in a closer layer to the NP surface, called “hard-corona” [23, 24]. Considering that these relevant for modeling novel NPs, in this work, it has been designed and performed a cost-effective assay that allowed to systematically decode the composition of hard- and soft- protein corona (in different biological fluids: human plasma, rabbit plasma and fetal bovine serum (FBS)) by centrifugation process and further proteomics analysis. IONPs and IONPs conjugated with biliar-acid-CisPt derivative [16] (IONP-Pt) has been evaluated by this strategy (Fig. 2B).

**Fig. 3:**
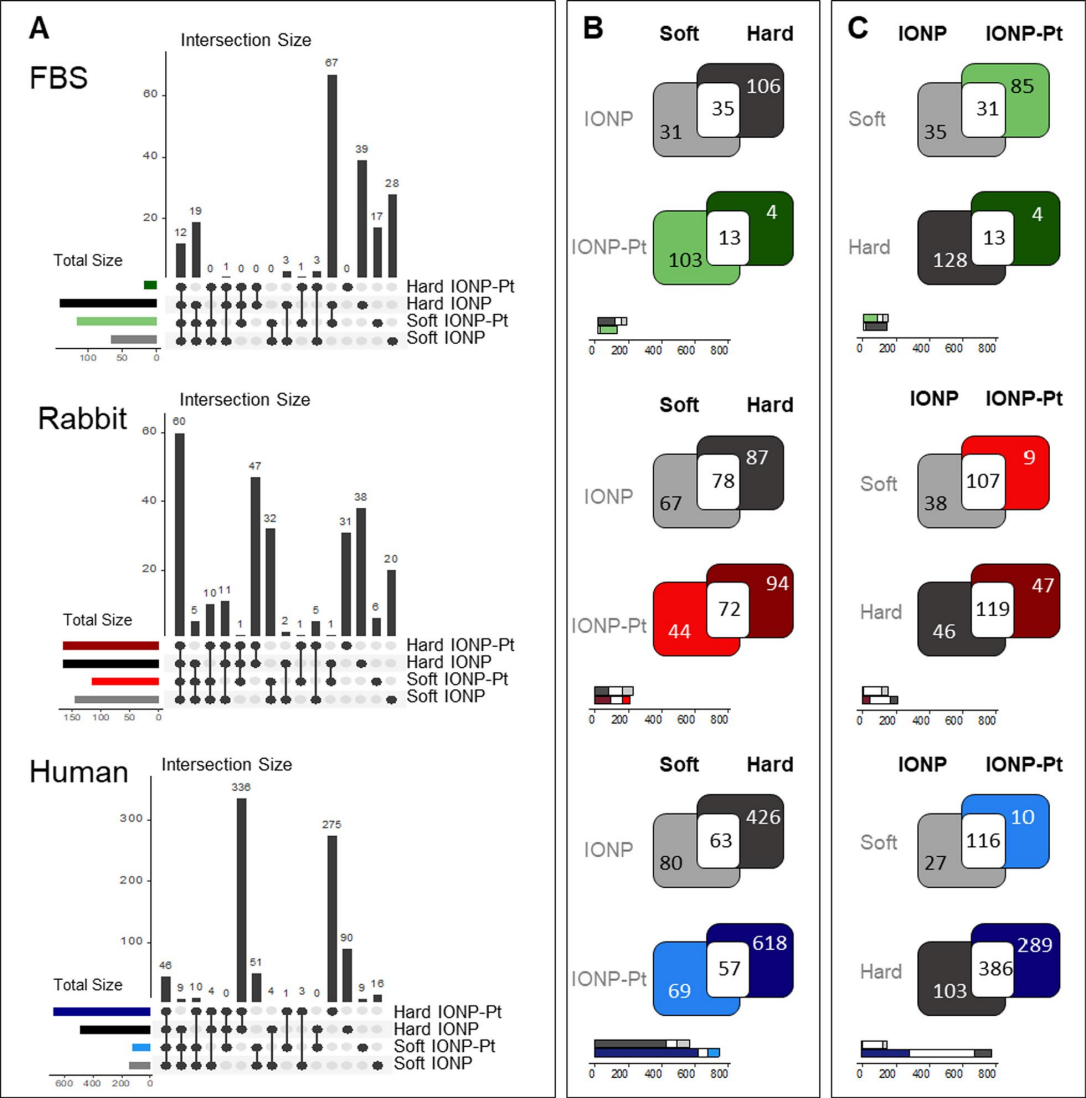
Overview and overlap analysis of different protein corona. **A** Bar plots displaying the number of proteins found in common between soft and hard corona of NP and NP-Cis-Pt across FBS, rabbit and human experiments. **B** Venn diagrams and stacked bar plots describing the overlap between soft and hard corona. **C** Venn diagrams and stacked bar plots describing the intersection between NP ± Cis-Pt

In Fig. 3, it is shown a quantitative comparative analysis about the number of unique proteins detected in the protein corona of each biological fluid. The overlap analysis revealed notable differences across the analyzed protein coronas at different proximal fluids; being reported a high number of identified proteins in human plasma compared with other studied proximal fluids. In particular, human IONP-Pt protein corona was > 5 and > threefold larger than FBS and rabbit-derived corona, respectively. Overall, the affinity protein corona displayed larger differences when comparing soft and hard corona (Fig. 3A and B) than when evaluating the presence or absence of Pt derivative conjugated to NPs (Fig. 3A and C). Newly, this trend is particularly conspicuous at human-derived protein coronas (Fig. 3, bottom row) where the highest number of identified proteins allows to observe the most remarkable differences between soft- and hard-corona. In a general overview, the fraction of proteins common to both -soft & hard- is quite relevant because it is highlighted a constantly presence in the final nano-drug conjugate. Although it the largest presence of proteins was determined in the human plasma-derived corona, according to the expected abundance of proteins in this fluid, the rabbit plasma provided a greater number of common proteins between the two coatings (Fig. 3, middle row). FBS-derived analysis resulted in the smallest numbers on proteins comparing with the other studied species. (Fig. 3, top row). However, in this case, the influence of the presence of Pt seems remarkable because 67 proteins identified at FBS-derived hard-corona, which were also detected at soft IONP-Pt. This is suggesting that the presence of Pt is modifying the interaction affinity of those proteins (Fig. 3, top row). The functional enrichment of the detected proteins provide a view of the type of protein binding in this case, where mostly of them are related to blood functions (coagulation, would healing…). Knowing that these factors are in the protein contain of FBS, it is understandable that the presence of Pt displaces these proteins towards the soft-corona, as these proteins interact with more affinity with the metal [25, 26] and do not reach to interact with the IONP surface (Additional file 1: Table S1). Finally, human protein corona reveals the largest differences between identified proteins at hard and soft coronas (Fig. 3, bottom row). Human plasma, on the other hand, presents much more diversity regarding the protein content. Thus, the number of proteins is higher and with more diversity between the layers of the protein corona as expected. When the NPs are conjugated with the Pt derivative, IONP-Pt, the human hard corona incorporates additional 275 different proteins (representing 40.1% of total proteins) (Fig. 3, bottom row).

**Fig. 4:**
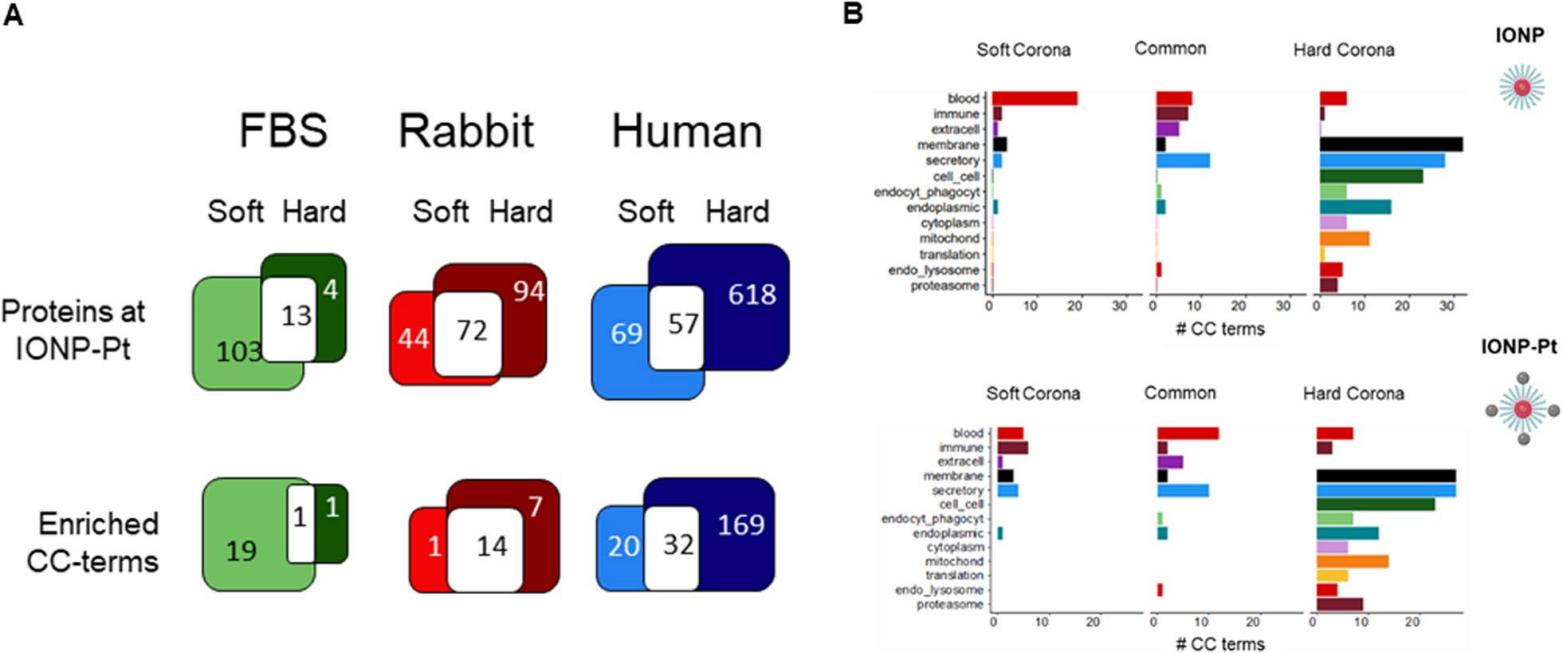
**A** Venn diagrams indicating total number of identified proteins at soft and hard corona at NP-Cis-Pt across the different animal models and total number of Gene Ontology-Cellular Component (CC) terms respectively enriched. **B** Bar plots summarizing most relevant Cell Component signatures enriched at human hard and soft corona proteomes with both NPs evaluated in this characterization

Number of identified proteins gives the prospect of a different dynamic behavior between species which is to be expected due to the diverse variety of biological content of plasmas; but also it is revealing the importance to evaluate the protein corona in all the proximal fluids involved in the development of nanodrug conjugates (from cell culture, animal model and preclinical). As well as, wide diversity on protein structures which directly influence in the interaction of protein corona onto the surface of NPs. This premise was also taken into account for the functional analysis of the protein as parts of protein corona (Fig. 4).

Cellular Component-Functional enrichment analysis reveals the distinct biological hallmarks associated to hard and soft corona in the plasma of species of interest (human, rabbit and bovine) (Fig. 4A). To characterize the biological hallmarks of the proteins found along the multiple protein coronas, two independent functional enrichment analyses of Gene Ontology-Cellular Component (CC) terms were performed separately for hard and soft protein coronas at each animal model (Fig. 4). Functional enrichment of Cellular Component-Gene Ontology annotations is a powerful tool to identify the most prominent macromolecular complexes represented in a protein set. In the same way, it brings relevant indications regarding the protein’s subcellular localization and thus, their biological roles.

As expected, due to the size of original protein set, the analyses of FBS and rabbit-derived protein coronas barely retrieved functional annotations (Fig. 4A). On this basis, the next analysis was restricted to human-derived protein corona. In order to summarize Functional Enrichment Analysis (FEA) results, the enriched CC annotations were classified into 13 groups representing the most relevant subcellular localization or processes. Finally, the CC-functional enrichment commonalities between soft and hard of IONP and IONP-Pt corona were assessed (Fig. 4B). The functional enrichment revealed notorious differences between hard and soft corona, both in total size and functional signatures (Fig. 4).

Interestingly, the functional annotations of identified proteins at soft corona are mainly related to macrocomplexes—commonly found at peripheral blood—as lipoproteins, platelet-coagulation C protein-related complexes or IgG and IgA immunoglobulin complexes as previously reported [tenzer2013] (Fig. 4B-left). Conversely proteins identified at hard corona and therefore, the NP stable interactors are drastically distinct (Fig. 4B-rigth). At hard corona proteome, identified proteins are involved in cell–cell contacts as integrins or cytoskeleton protein (ie. actin, microtubles, among others). Likewise, hard-corona proteins prompt to be particularly and significantly enriched in processes such as endocytosis, phagocytosis and intracellular trafficking (including COPI or clathrin-coated vesicles and endomembrane system complexes). Hence, these results are suggesting the activation of late endocytic-NP digestion processes because it is observed enriched proteins involved in endosome and lysosome-related proteins together with proteasome core and regulatory complexes. These observations correlate with the normal distribution of proteins in plasma, where proteins in highest concentration (such as the function “blood”) appears in soft-corona while the proteins in lower concentration conform the hard-corona. The influence of the platinum complex is also noteworthy, which promote proportional modification on the functions associated to soft-corona and not those relative modifications to the hard-corona as might be expected from the dynamics of the protein-corona formation. These changes in the different composition of the protein layers close the NPs as a consequence of the functionalization must be considered when evaluating and establishing the biocompatibility of each of the modifications that are established in the nanoconjugates.

Apart from general functional enrichment, here it is also relevant to explore the differences in distribution at cellular compartment between soft- and hard-protein corona. At Human Protein Atlas (HPA) according to the tissue of origin, the proteins observed are related with blood secreted proteins, extracellular matrix (ECM), and other tissues as lung, epithelium or endothelium. Also, it provides proteins involved in secretion pathway frequently found restrained at organelle lumen or membrane. By this, it is obtained a global view of the histological origin of the proteins related to the human-derived corona (Additional file 1: Fig. S1). While soft-corona, in both situations ± Pt derivative functionalization, is composed by large percentage of blood secreted proteins; hard-corona proteome barely maps into a global secretome universe (Additional file 1: Figs. S1 and S2). Around 80% of detected proteins at hard- corona have not been previously reported at HPA secretome; it was evaluated the most frequent tissue of origin by assessing their RNA expression levels at 33 tissue-specific RNA-Seq datasets (also retrieved at HPA database) (Additional file 1: Figs. S1 and S2, Table S2). For 410 and 570 proteins found at hard IONP and IONP-Pt corona, respectively, approx. 90% were identified in at least one of the 33 tissue-specific RNA-seq data sets. Disregarding differences in total number, the tissue of origin most over-represented, (hard-corona in both NP and functionalized NP), are bone marrow, tonsil, esophagus and colon. On the other side, as it may be expected that less frequent tissues of origin correspond to sexual tissues as endometrium, testis, prostate or fallopian tubes (Additional file 1: Figs. S1 and S2, Table S2).

### In vitro cytotoxicity of multi-functional NPs

**Fig. 5:**
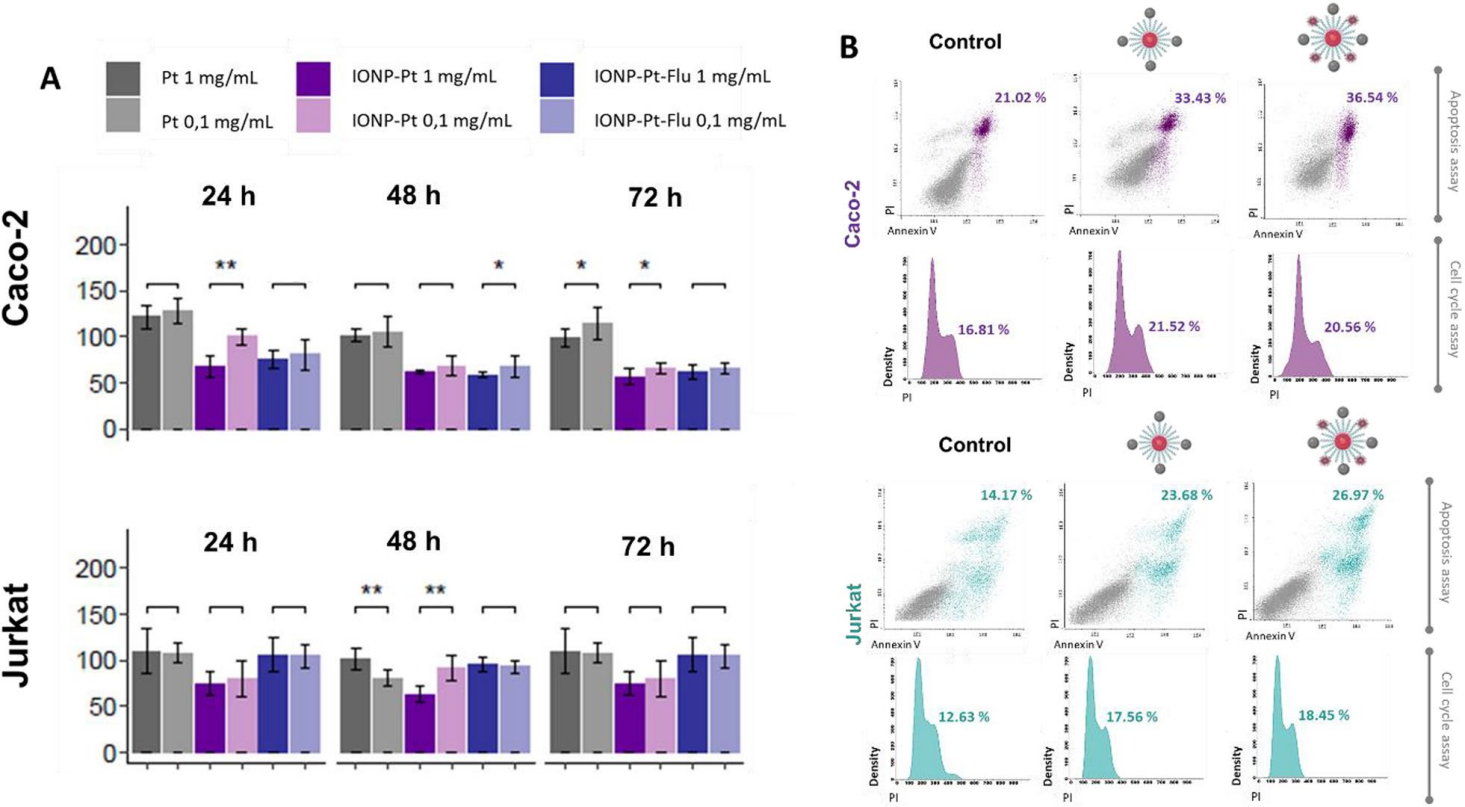
**A** Viability assay performed in Caco-2 and Jurkat cancer cell lines using Pt precursor, IONP-Pt and IONP-Pt-Flu at different concentrations (1 and 0.1 mg/mL) incubating for 24–72 h. Results are reported as the mean number of live cells relative to the control (vehicle) from three independent experiments (significantly differences p * < 0.05, ** < 0.01). **B** Flow cytometry results (apoptosis and cell cycle assays) of NPs tested (IONP-Pt and IONP-Pt-Flu) for 24 h in Caco-2 cell line and Jurkat cancer cell lines at 0.1 mg/mL. Percentage of apoptotic cells (annexin+) and G2/M cells of each experiment are shown in the corresponding diagram as the mean of two independent experiments for each condition

The cytotoxicity of this novel NPs, it is another critical aspect in the characterization of the novel multi-functional nanodrugs, as these novel NPs are containing a fluorochrome covalently coupled to the surface. The main goal was determining whether this functionalization with the fluorescence dye may affect the release of Pt from the initial structure, or the fluorophore coupling could affect to the biocompatibility of the IONP (Fig. 5). Then, it has been explored the cell viability of two tumoral cell lines (colorectal cancer (Caco-2) and T lymphoma (Jurkat)) as models and studied the effect at different concentrations (1–0.1-mg/mL) of IONP-Pt and the multi-functional NP with a fluorophore (IONP-Pt-Flu). In Fig. 5A, it is observed a similar cell viability in both studied human cell lines. In the case of IONP-Pt, cell viability resulted similar to previously reported studies which described the compound triggers cellular instability reflected with a large increase in cell viability [16]. Some slightly differences have been observed in both tumor cell lines. At first glance, it can be seen that the use of IONPs improves the activity of the platinum compound as it shows less cytotoxicity than the IONP conjugate. In general, it has also been shown that there is a slightly higher cytotoxicity in Caco-2 cells than in the Jurkat tumor cell line. In addition, in the Caco-2 tumor cells, it can also be seen that there are more similarities in the behavior of IONP without any influence from the degree of functionalization. About Jurkat tumor cell line, it can be observed that there are certain discrepancies in the behavior of the functionalized IONPs at different incubation times (Additional file 1: Fig. S3). This is due to the oxidative stress-inducing effect mediated by Pt-derivatives as a cytotoxic mechanism [27], and it was reported in previous studies of this kind of platinum-IONPs [16]. In a similar way, it is observed that the fluorochrome did not affect the biocompatibility of IONPs as the detected cytotoxicity did not significantly changes when cells were exposed under both conditions (Additional file 1: Fig. S2). To further characterization of biocompatibility of novel multifunctional NP, cell cycle and apoptosis were determined by flow cytometry (Fig. 5B). IONP-Pt and IONP-Pt-Flu were tested (0.1 mg/mL) at 24 h of incubation using a combination of annexin V and propidium iodide (PI) in order to outline the profile of drug-induced cell death. In addition, the cell cycle analysis will provide a view of possible effects at the different phases. Platinum derivatives induce a cycle arrest in the G2/M phase preventing the correct DNA replication due to their crosslinking effect and formation of adducts between the DNA base pairs [28]. As depicted in Fig. 5B, both tested cell lines show a different behavior in the cell cycle; however, it appears that in both cases the NPs induce an equal response. About apoptosis, it is slightly increased in both cell lines under the assay conditions, which are set up according to obtained results about the cell viability experiments (Fig. 5A). Bearing in mind the NP functionalization, no significant variation is observed between IONP-Pt and IONP-Pt-Flu in either of the two tested cell lines. Regarding the cell cycle analysis, an increment of cells in G2/M phase was observed in the Caco-2 cells with both studied NPs; and similarly, in Jurkat cells. Overall, these results perfectly match with expected mechanism of action for Pt derivatives. In summary, the preservation of the apoptotic response as well as minimal variations in the cell cycle suggest that the multifunctionalities of the IONP-Pt-Flu does not have an impact on drug release from the IONPs nor a variation in the pharmacological response.

### Proteomics approach for deciphering the intracellular biology of multi-functional NPs

Further analysis, it is focused on the exploration of intracellular pathways by systematic proteomics characterization. Intracellular study of the activity carried out by the NPs is necessary to confirm that the Pt derivative has been successfully released and also to identify the expected and novel protein targets. Deciphering the perturbations in the signaling pathways linked to this compound are key to know if there are modifications in the release process of the pharmacological load of the NP. Also, they are useful to know the implications that the functionalization of the NP may cause in the intracellular signaling pathways on the cells of interest.

**Fig. 6:**
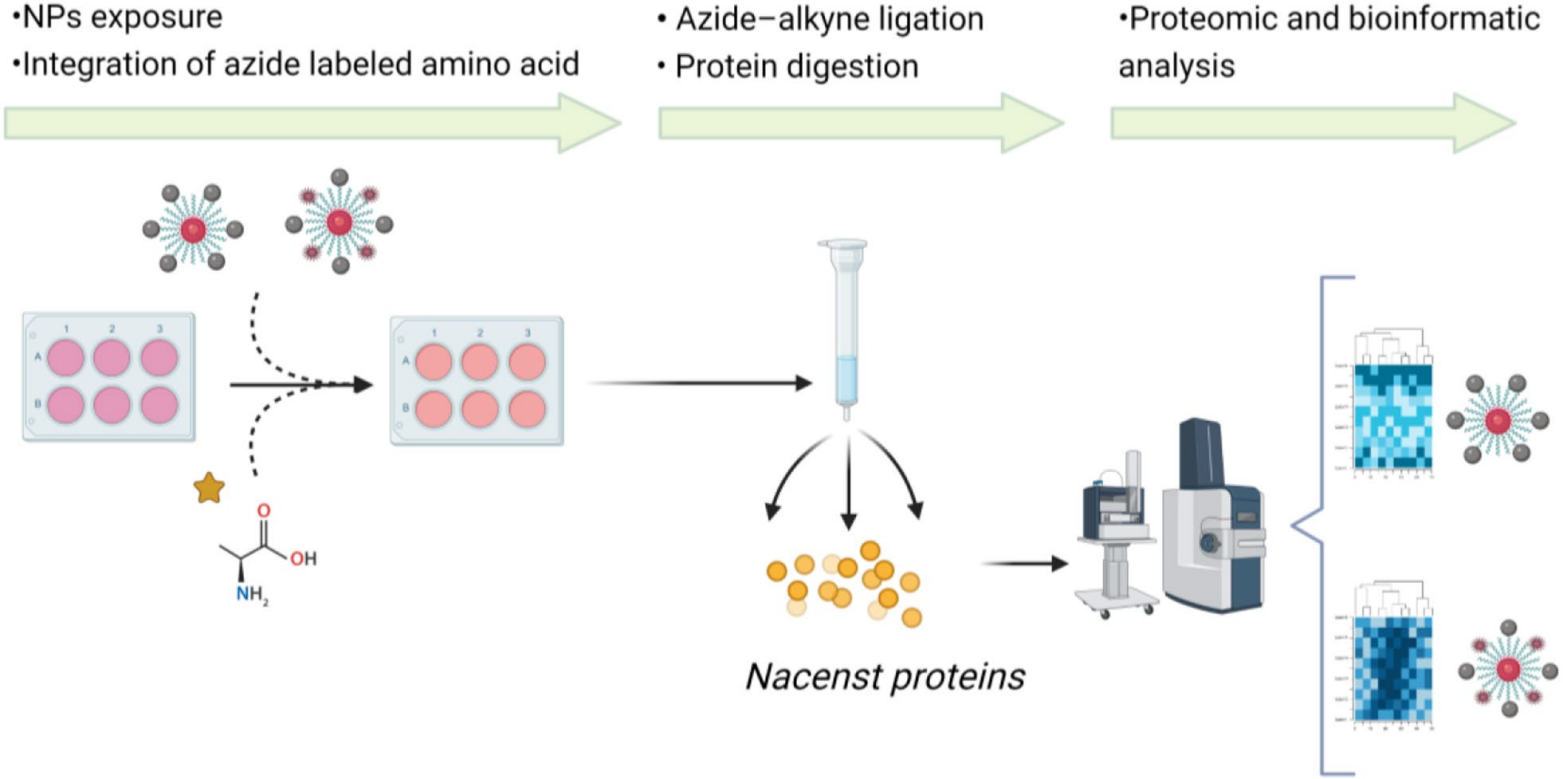
Workflow of proteomic analysis performed to decipher intracellular pathways related to IONP-Pt and IONP-Pt-Flu

Proteomics is a precise and suitable technique to understand the behavior of cellular stimuli, providing a general and detailed overview of the proteins involved in signaling pathways, organelle activation or cellular communication processes; despite the potential of proteomics approaches, it is required to design experimental procedures focused on the analysis of targeted proteins or proteome landscape. Here, in this study, a technique based in protein labeling with non-canonical amino acid azidohomoalanine (AHA) provided the ability to specifically and selectively detected of de novo synthesized proteins uniquely related to intracellular pathways triggered by NPs. Tumoral cells lines (Caco-2 and Jurkat) were exposed to the functionalized NPs including in the cell culture medium AHA amino acid that was integrated into the nascent proteins, as described in materials and methods section. Further bio-orthogonal chemistry between the amino acid and a copper-catalyzed azide–alkyne ligation allowed isolation of tagged proteins (which are newly synthetized). A column previously prepared with a resin functionalized with an alkyne derivative allowed the specific capture of the labeled proteins. This methodology also allowed in situ trypsin digestion of the newly synthetized proteins, directly providing trypsin digested and purified peptides (Fig. 6).

**Fig. 7:**
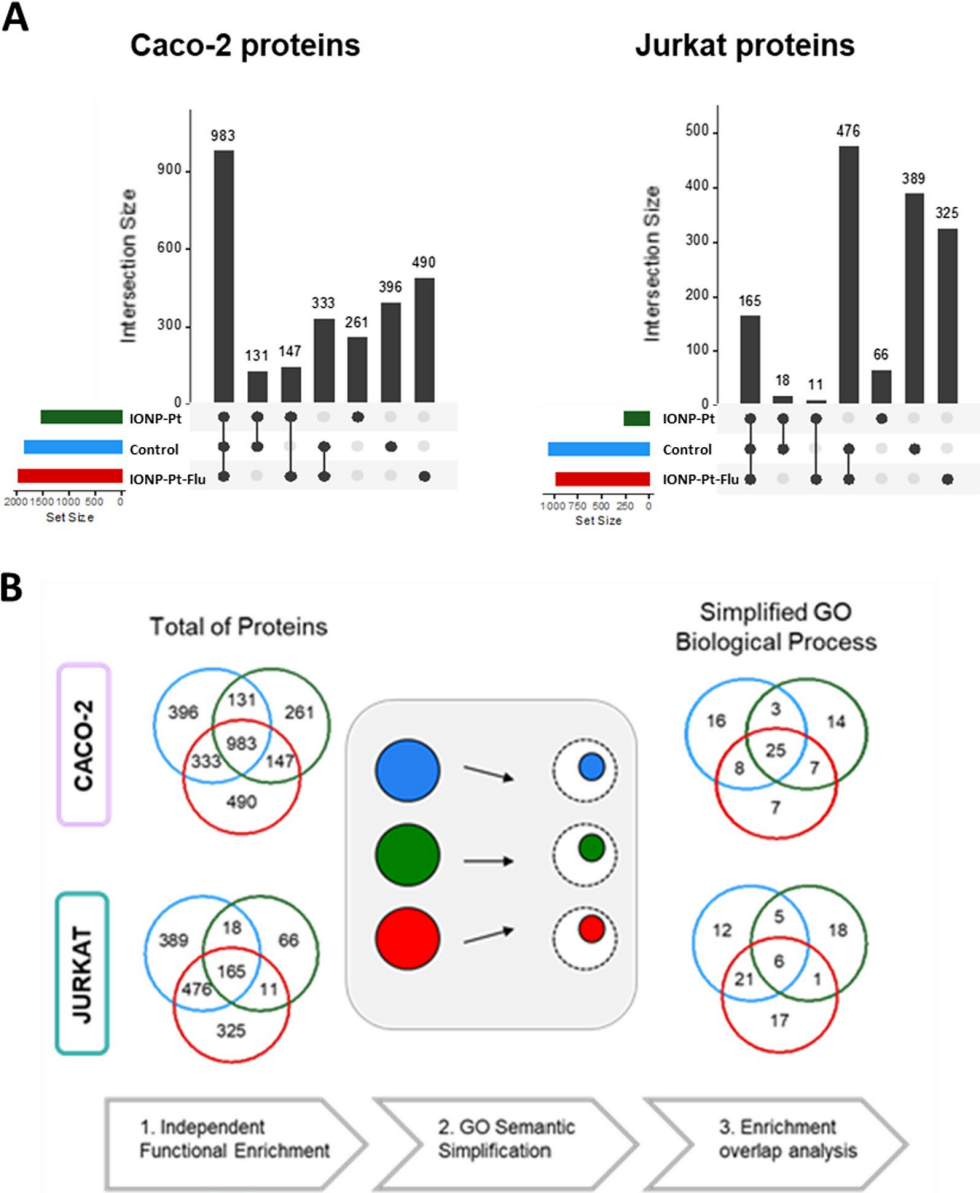
**A** Functional characterization pipeline illustration and summary. Left Venn diagram depicts the common proteins synthesized at different NP conditions and right venn diagram the respective functional enrichment similarities after functional semantic simplification. **B** NP-induced newly synthesized proteome overview and overlap analysis. Bar plots and venn diagrams showing the number of proteins commonly synthesized at control conditions and NP-Cis-Pt or NP-Cis-PT-FITC presence at the experiments conducted on Caco-2 (**A**) and Jurkat (**B**) tumor cell lines respectively

At first glance, the global proteome is analyzed in order to distinguish differences across several studied tumoral cell lines and NPs of interest. Attending of the total number of proteins in each condition and cell lines, a significant quantitative difference can be observed in the total number of proteins depending on the cell line, being in general twice as many proteins detected in the colorectal cancer cell line (Caco-2) than in the hematological (T cell leukemia) tumor cells line (Jurkat) (Fig. 7A), (according to the functional enrichment of Gen Ontology-Biological Process (GO-BP)). This difference becomes even more remarkable when observing the effect triggered by the IONP-Pt, where in the Caco-2 cells a fourfold higher number of proteins were detected than in the Jurkat cells (Fig. 7B).

Considering this general analysis provided by the high-content proteomics characterization, a simplification of the functional enrichments has been carried out to facilitate the biological outcome from cell signaling pathways comparison. The functional enrichments were simplified into broader functional groups according to Lin’s semantic similarity > 0.8 using REVIGO method [29]. The simplified functional commonalities were assessed only for the proteins de novo synthesized at Caco-2 and Jurkat cells at different IONPs conditions and controls (Fig. 7C right). In this analysis, the differences between tumor cell lines are minimized. Nevertheless, this proteomic approach allows us to discern certain differences in the pharmacology of the tumor cell lines. Interestingly, differences between cell lines were found in the most functionalized NP (IONP-Pt-Flu), where a higher number of functions (more than double) were detected in the hematological tumor line compared to the Caco-2 cell line. This phenomenon is explained by the different functional specialization of the cells. The adaptive immune system is composed of highly-specialized systemic cells. The functional specialization is patent when considering the total number of proteins identified in the assays and the respective universe of functions annotated in GO repository for the proteomes of Jurkat and Caco-2 cells. We have corroborated that the initial functional annotations present almost the same hierarchical depth; thus, the semantic simplification did not bias the results in Additional file 1: Fig. S4. On this basis, we claim the difference observed in functional enrichment of the two cell lines reflects the difference in the level of functional specialization between Jurkat and Caco-2 cells.

**Fig. 8:**
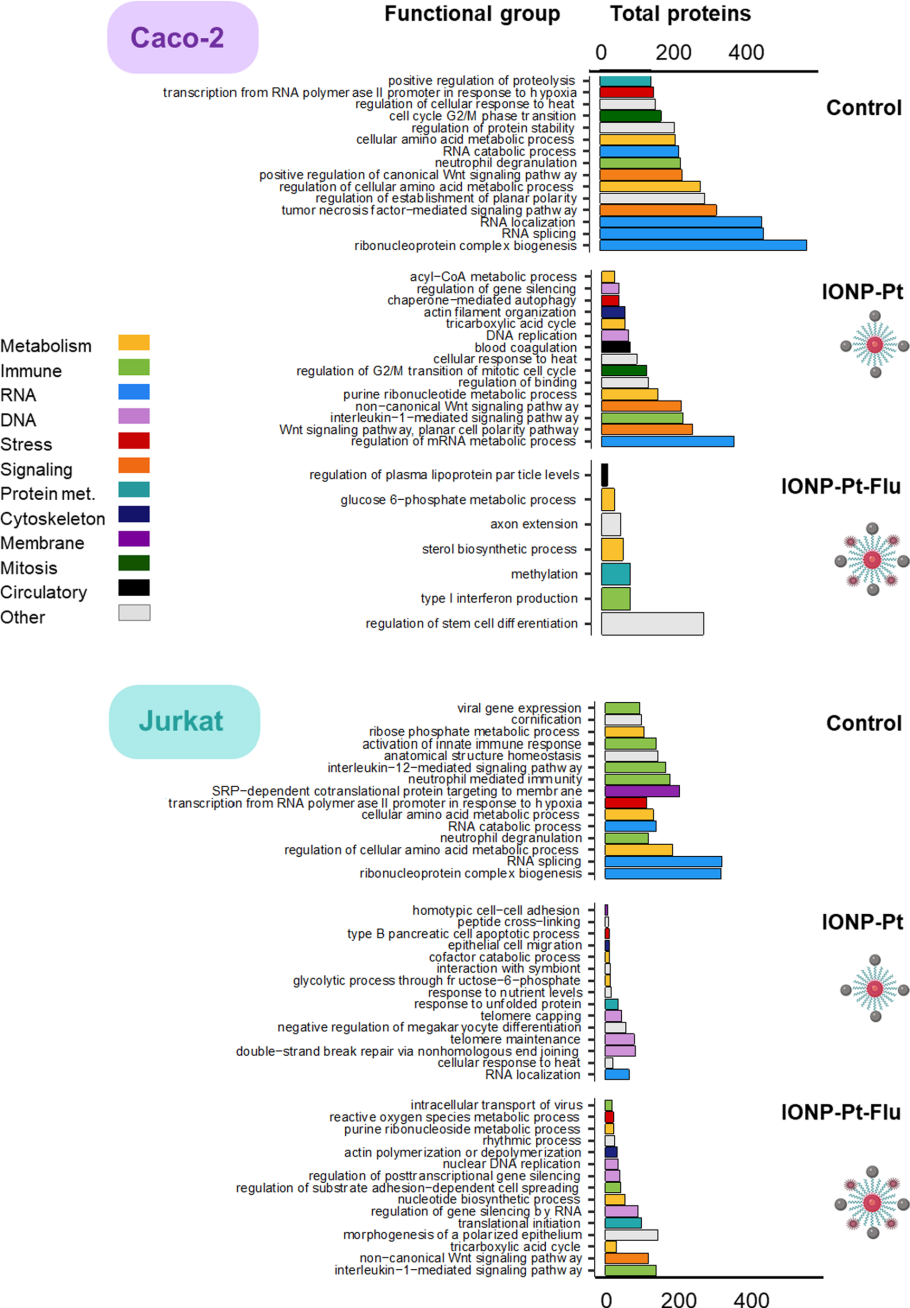
Analysis summarizing functional simplification in Caco-2 and Jurkat cell line. Each function color indicates the resulting functional group according to REVIGO method

The potential pharmacological features of these novel nanodrugs might be described by the selective and specific qualitative functional enrichment analysis of the newly synthetized proteins in studied conditions. Moreover, it was possible the one-step and simultaneous global analysis of intracellular effects related to the multi-functionalities of the IONPs. The analysis of the 15 most important biological functions in the Caco-2 control line are in accordance with the reported and expected for this type of solid tumor (Fig. 8, Additional file 1: Table S3). It has long been known that in colon cancer, there is an alteration of the anaphase-promoting complex (APC) involved in the control of the transition of the cycle to G2/M phase. This also has implications in the activation of the Wnt pathway [30]. Amyloid-beta clearance [31], regulation of folding and stability of proteins as wells as RNA metabolism are known to be disrupted in colorectal cancer [32, 33]. When cells were treated with IONP-Pt, the protein expression profile has changed according to the effect of Pt-derived compound conjugated with the IONP. The activation of cellular functions such as “DNA replication” (including proteins such as TOP1A) are consistent with the pharmacological effect of Pt as a DNA crosslinker. Also interesting is the activation of the “purine ribonucleotide metabolic process” function where the PARP is significantly observed which is a protein related to mechanisms of repairing DNA damage. Likewise, the “chaperone-mediated autophagy” function is related to the endoplasmic reticulum stress mediated by platinum compounds as it reveals the appearance of the protein HSP90. Regarding the multifunctional IONP, general cellular processes related to Rho GTPase family are observed. Other previous functions of the analysis are observed (both actin and axon are mediated by the Rho GTPase family). Also, FEA is not highlighting any other pathways that can be related to cytotoxicity directly related with the multifunctional properties of these IONPs. Thus, by these studies, it is confirmed, in Caco-2 cells, the release of Pt compound which makes feasible the specific molecular targeting, and it is also observed that the influence of the multi-functionality with respect to the pharmacological effect is not affecting a successful drug disposition.

The analysis in the hematological cell line yielded very similar conclusions about the behavior of multifunctional NPs and the pharmacological effect of the platinum derivative. The basal state of the analyzed Jurkat cells as a control condition resulted in the appearance of 15 most relevant functions related to activation of the immune system as well as alterations in cell replication. These functions are in accordance with the nature of cell (T lymphocyte) and the tumoral pathology (Fig.8, Additional file 1: Table S3). In cells exposed to IONP-Pt, the function related to non-homologous recombination of damage repair underlined, which is directly linked to the action of the platinum compound. According to the specific proteomic data obtained, in this cell line it is due to activation of histones as well as DNAPKcs kinase. The oxidative stress mediated by the Pt compound is reflected in the “ER-nucleus” function where EIF2S1 appeared. Also highlights again HSP90 protein in this group; being both proteins known as key factors in response to cellular stress. Similarly, these proteins are also part of the function “response to unfolded protein” which is also related to oxidative stress. Regarding the alkylating power of platinum derivatives, in this case also analysis provided “peptide crosslinker” as a leading function. Coupled with the DNA alkylation, this process represents a cytotoxic mechanism of platinum derivatives. As for the IONP-Pt-Flu, results linked to DNA damage were also obtained, such as the impairment of nucleotide synthesis where PARP appears detected. Likewise, the mini-chromosome maintenance (MCM) proteins family also appear within the “nuclear DNA replication” function, which are responsible for the formation of the nuclear replication complex. Likewise, HSP90 participates in the regulation of reactive oxygen species. This more specific analysis of the different conditions in the Jurkat cells has resulted in a correlation of functions in accordance with those proposed for the colorectal cancer assay (Fig. 8).

The main functions associated with the drug are maintained independently for both IONP-Pt and IONP-Pt-Flu. Therefore, a low number of significantly detected proteins is maintaining a high number of similarly functions, which mainly be related to redundant, pleiotropic, cooperative and synergically effect of these proteins on the immune system and its response. Bearing in mind the results from functional proteomics characterization, it is observed a correct drug delivery to the molecular target as far as the IONP is concerned and no significant influence of the fluorochrome is appreciated that could interfere in the pharmacokinetic process. In summary, the proteomic technique chosen to decipher the intracellular signaling of these novel multi-functionalized IONPs can be considered very suitable, since it has been able to differentiate the implications of each of the NP components. Likewise, the bioinformatic analysis performed on the data has been highly enriched. General and detailed analytical results have been tightly correlated between cell lines as well as coherent with the studied nanotechnological platform.

### In vivo evaluation of NPs activity

To conclude with the integral characterization of the NPs and their biocompatibility, an initial in vivo approach has been carried out. IONPs and CisPt precursors were tested using rabbit as an animal model. In this case, a liver tumor by implantation of fragments of VX2 tumors (squamous cell cancer line) were developed to test the efficacy of platinum compounds and the corresponding nano-drugs conjugates. For this test, apart from BilPt (biliar platinum complex, see Fig. 1) and IONP-Pt, [Pt(Cl)_2_(NH_3_)] cisplatin compound (CisPtCl) and the corresponding IONPt-CisPtCl (positive controls), IONPs (negative control) were also included in the screening.

**Fig. 9:**
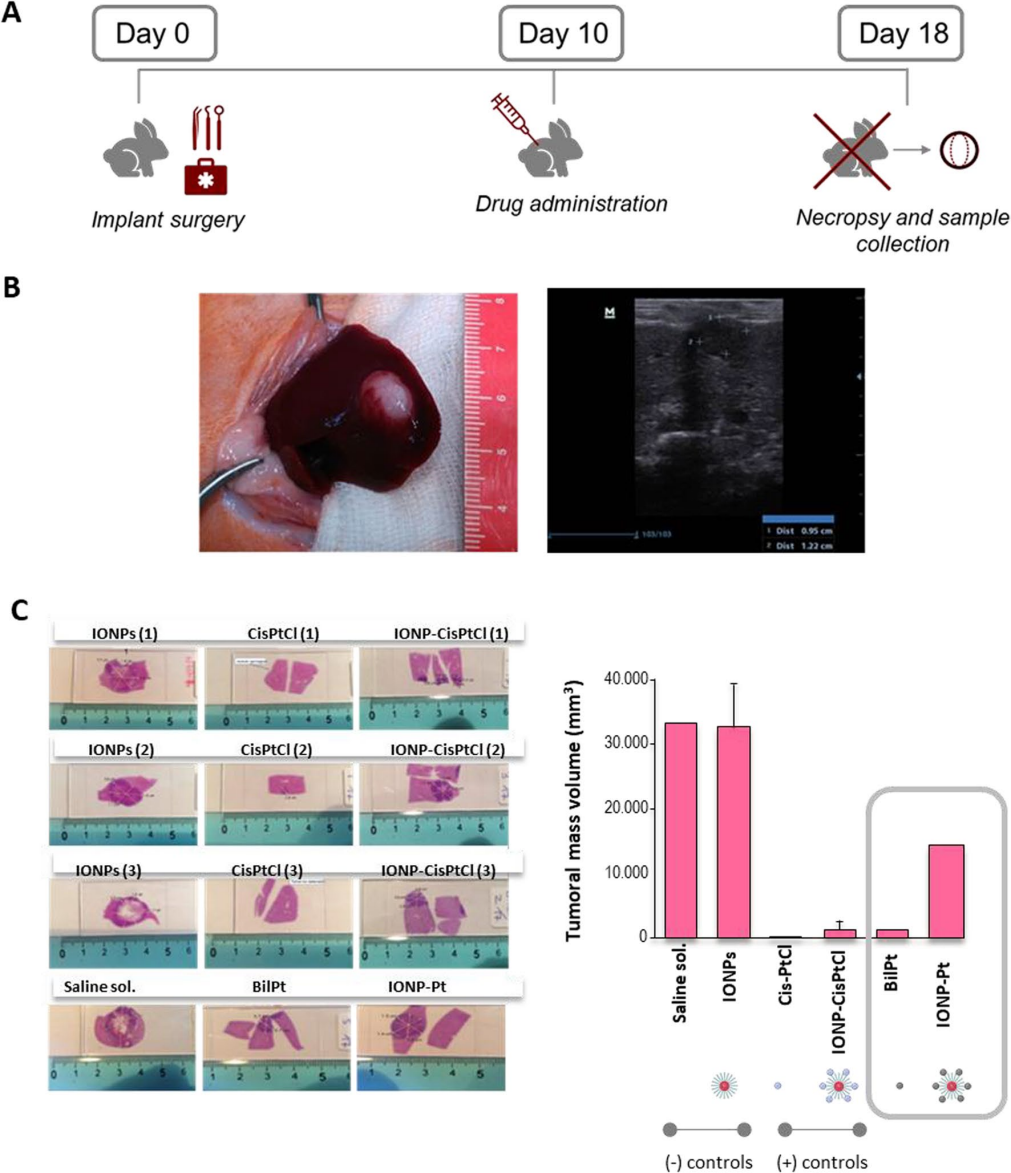
**A** Chronology of tumor implantation and pharmacologic treatment. **B** Image obtained during the necropsy of one patient N its corresponding image obtained the same day. It can be seen how the tumor mass protrudes from the hepatic parenchyma coinciding with the ultrasound image. **C** Left, Images of the histological sections of the implantation area of the different animals, as well as the measurements taken on them (right). Right, tumor mass volumes (mean) obtained during the necropsies

When the animals were intervened, the tumor mass was allowed to grow until 10 days later, then treatment with drugs was started. After 8 days of treatment initiation, the animals were sacrificed. The localization and follow-up of the tumor was carried out by periodic measurements with ultrasound scans, finally verifying the correspondence between the images and the tumor after excision of the tumor mass (Fig. 9).

Histological examination of the samples yielded interesting results about the compound studies and the corresponding nano- drugs conjugates (Fig. 9C and Additional file 1: Table S3). The saline solution used as vehicle (negative control) as well as the administration of the naked/plain IONPs (without functionalization) resulted in tumors of large volume and similar size (Fig. 9C). This demonstrates the function as drug carriers of these NPs, with no other cytotoxicity per se. The CisPtCl used as a positive control resulted in the appearance of a minimal tumor mass or the absence of tumor in the animal (Fig. 9C). Treatment with the standard CisPtCl was able to reduce tumor mass to a volume 40-fold less than the negative control (Fig. 9C). Similar reduction was observed when the BilPt was administered, indicating an outstanding efficacy of this product. When the standard CisPtCl was encapsulated in the IONPs, similar reductions were found with respect to the product alone, thus indicating the effectiveness of IONPs as suitable drug carriers. The administration of IONP-Pt did not achieve the values of the positive control nevertheless, it was still able to reduce the tumor mass to one third of the mass developed without treatment (Fig. 9C).

## Discussion and conclusions

In this study, a full complete characterization of NPs by an integrated workflow covering from NPs multi-functionalization to in vivo animal model has been performed. This characterization is based on conventional approaches for biocompatibility (such as cell viability, cell cycle…) and to proteomics approaches for analysis of IONPs-microenviroment interaction and de novo intracellular proteins due to IONPs. The successfully design and development of multifunctional IONPs that simultaneously carried an antitumor drug together with a tracing system (flourochome), it seems to open up many possibilities for their application in targeted and personalized antitumoral therapy [18]. Similarly, the fact that a high diverse variety of IONPs functionalization does not alter their properties as a drug delivery system in vitro, or reduce the antitumoral capacity, which is suggesting as an advantage the versatility of IONPs as potential targeted onco-therapeutical agents. The multiparametric biological characterization of these IONPs has corroborated this multiple conjugation capability with their confirmed and verified biocompatibility.

Regarding the exhaustive analysis of protein corona in different proximal fluids (human serum, rabbit serum, culture media) is probing that IONPs display differential behavior according to each particular microenvironment; hence, it is critical to evaluate always the protein corona in all the possible conditions in order to properly define the IONPs biocompatibility [17]. Proteomics approaches have demonstrated to have a very interesting application in protein binding to NPs. As described in the recent report of Tao et al., where some of the proteins found in this work, are also described in the protein corona of these novel IONPs. As has been observed in this same work, there is a great difference between the protein corona in the different fluids studied [34]. In this sense, a difference has also been found between the activity observed in the in vitro studies of this and previous reports [16] and the in vivo studies proposed here. Future investigations between in vivo and in vitro NP structure–activity relationship should undoubtedly consider the protein corona to make the translational breakthrough of these nanoconjugates.

As expected, the differential protein profile (protein content in all the analyzed plasmas), was reflected in the analysis. However, these differences, far from being a drawback for our study, confirm the relevance and importance of following the dynamics of change between species in order to carry out more rational biocompatibility studies and to be able to establish more conclusions between in vivo and in vitro studies, which are required and critical in all the biomedical and preclinical studies.

Proteomics also provided us great results in the description of the intracellular activity. Although there is a great difference at the specific protein level between conjugation with the drug, regarding the altered intracellular protein profiles, it seems that the cytotoxic capacity is not dramatically affected on the targeted studies cells according to the bioinformatic approaches applied. According to previous studies on similar IONPs [16], it has been reported that direct labeling with a fluorescence dye, it is maintaining the antitumoral capacity of the NP-conjugated cisplatin-derived drug. The usual mechanisms referred to this compound have been found in each of the conditions studied without other accessory alterations that could be attributed to the increase of components in the final NP. In this sense, following the high-throughput proteomic, the majority of de novo synthetized proteins are directly related with immune functions, as also occurs in protein corona. Then, the immune system activation links and the differential intracellular protein profiles are involved in mechanisms encompassed in the immunogenic cell death (ICD) [35], cell death mechanism links to platinum-derived compounds. The ICD concept has been identified as cell death mechanisms that elicits complete immune responses when certain intracellular process such as oxidative stress (very marked under the conditions tested in this work) and the release of damage-associated molecular pattern (DAMPs) reflected here with the activation of HSPs detected in proteomics assay [36, 37]. These facts promote of further and future studies in which not only the pharmacological effect of these compounds is studied, but also their immunogenic role. The final in vivo assays performed in this report complete the biopharmaceutical profile of the bile acid-conjugated platinum compounds and the novel IONPs, being pharmacologically active and displaying their cytotoxic capacity. Once again, the relevance of integrating biocompatibility data (protein corona), in vitro studies and animal model results is highlighted.

Drugs and nanoparticles must be understood in their broadest sense (chemical, pharmacological, environmental…). In this work, we have aimed to study in a wide and systematic manner the contributions that the combination of multifunctionalities in NPs could improve the final drug conjugate. Among that, it is also focused in to understand the quantitative gap between in vitro and in vivo studies, and between species. The studies carried out provide us with a contribution of the NPs in the in vitro assays compared to the platinum compound. Although this contribution is not reflected in vivo, the biocompatibility of the IONP with the animal model and with the reference CisPt can be observed. The advantages of IONPs are well-known and reviewed [38–41]. Another positive aspect of encapsulating CisPt derivates, it is that the efficiency is not being modified. Despite of being encapsulated, it is being reported that these IONPs are suitable for drug delivery systems; in addition, the side effects seems to be lower than those caused by free CisPt, which will have a more extensive and systemic distribution. In general, the characterizations provided in our work go beyond the usual assays and reflect the importance of a correct extrapolation of results between species and taking into account cellular targets.

Finally, we would like to provide a wide-open perspective on the coating implications that can overlay nanoparticles. In this case, we have performed a high-throughput analysis of the protein layer, but other high-throughput methodologies (i.e.. genomics, lipidomics, metabolomics,…) must be taken into account to give translational application; such as, the metabolic corona is another layer to study, coexisting with the protein corona [42]. Other biocompatibility studies have also described the lipid corona [43]. These authors describe the implications of this lipid layer on the adsorption of NPs. It is demonstrated that the study of biocoatings (in the proximal fluids of interest) in NPs is essential for the complete description of the biopharmaceutical properties. Taking into account these perspectives and including genetic variations that may also affect the efficacy of NPs, it is demonstrated that nanoscience in drug discovery needs an integrative view of all the emerging omics perspectives.

It can be concluded that in this work a complete study has been designed and carried out from the chemical design of novel nanodrugs based on multifunctional IONPs with a complete description of their biocompatibility and pharmacological properties at both levels of interest, in vitro and animal model, which are required to speed up the screening for biomedical applications, mainly in personalized medicine.

## Experimental section

### Materials

#### Chemicals

Iron (II) chloride tetrahydrate (FeCl_2_ 4H_2_O, 98%), Iron (III) chloride (FeCl_3_, 97%) Copper (II) chloride (CuCl_2_, 99.999%), methyl 2-chloropropionate (MCP, 97%), Rhodamine B (≥ 95%), Succinic anhydride (98%) were supplied by Sigma Aldrich and used as received. 4-vinyl pyridine (4VP, Aldrich, 95%) was distilled under vacuum and stored at 5 ºC. Copper (I) chloride (CuCl, Aldrich) was purified with stirring in acetic acid, filtered, washed with ethanol and diethyl ether, and stored under vacuum. Methoxy poly(ethylene)glycol acrylate (MPEGA, Mn = 480 Da, Aldrich) was filtered through a pad of basic alumina before use. Commercially available Poly(ethylenglicol) methacrylate (PEGMA, Mn: 360 Da, Aldrich) was purified before use as describe in ref [12] in order to remove the dimethacrylates and diols impurities present in the commercial monomer (Mn: 390 Da, calculated by NMR after purification). Tris [(2-pyridyl) methyl] amine (TPMA) and Tris[2(dimethylamino)ethyl]amine (Me6-tren) were prepared following literature procedures [12, 16]. The synthesis and characterization of Rhodamine- poly(ethylenglycol) methacrylate (Rhod-PEGMA, Mn: 940 Da) and the amphiphilic block copolymer P4VP-b-P(MPEGA-co-RhodPEGMA-co-carboxylicPEGMA), (poly(4-vinylpyridine)-block-poly(methoxy poly(ethylenglycol) acrylate-co-Rhodamine poly(ethylenglycol) methacrylate-co-carboxylic poly(ethylenglycol) methacrylate) (Mn(NMR: 16 k Da), used in this study for the coating and functionalization of the of iron oxide nanoparticles (IONPs), has already been previously reported [16].

#### Biologicals

Annexin V FITC and PI, and annexin V binding buffer 10× were provided by Immunostep SL. (Salamanca, Spain). Dimethyl sulfoxide (DMSO) was obtained from Merck (Darmstadt, Germany). RPMI 1640 medium with l-glutamine, DMEM medium with l-glutamin, bovine serum albumin (BSA), trypan blue (TB), urea, and 3-(4,5-dimethylthiazol-2-yl)-2,5-diphenyltetrazolium bromide (MTT) were purchased from Sigma (St Louis/MO, USA). RC DC™ Protein Assay was purchased from BioRad (Hercules/CA, USA). 96-well plates, Coomassie Brilliant Blue solution, Click-IT™ AHA (l-Azidohomoalanine) and Click-iT™ Protein Enrichment Kit, for click chemistry capture of azide-modified proteins were obtained from Thermo Scientific (Rockford/IL, USA). Cycloscope™ Reagent for cell cycle analysis was purchased from Cytognos (Salamanca, Spain). Heat inactivated fetal bovine serum (FBS), l-glutamine, penicillin–streptomycin (P-S) and 0.25% trypsin–EDTA and DMEM, high glucose, no glutamine, no methionine, no cystine were purchased from Gibco®(Gran Island/NY, USA). 12-well clear flat bottom plates were purchased from Corning (Corning/NY, USA).

### Evaluation of protein corona

The protein corona assay was carried out to determine which proteins adhered more or less strongly (hard- or soft-corona) to the NPs, evaluating finally by LC–MS/MS. To quantify the proteins, RC DC™ Protein Assay was used following manufacturing’s instructions. Hard and soft protein corona determination: Based on previous reports, protein corona was isolated by centrifugation assays [44]. Briefly, NPs (100 μL) were exposed to undiluted Human and Rabbit plasma, as well as Fetal Bovine Serum (500 μL of each sample) and incubated for 1 h. After that time, samples were centrifuged five times (12,000 rpm, 10 min), with PBS (600 μL) and saving the supernatant in each time. Finally, the pellet was also saved on 50 μL of PBS.

### Protein quantification and SDS-PAGE separation.

Protein samples were quantified by using a modified Lowry protocol (RC/DC Protein Assay, BioRad, CA, USA) following the manufacturer’s instructions. A total of 30 μg proteins were run on a 4–20% gradient SDS-PAGE gel under reducing conditions. Gels were stained in a 0.5% (w/v) Coomassie Brilliant Blue solution and stored at 4 °C in an aqueous solution containing 1% (v/v) acetic acid, until analysis. In-gel digestion and nanoUPLC-MS/MS analysis. Each gel lane was cut into 5 fragments and digested with trypsin following the method of Shevchenko et al. [45] with slight modifications. Briefly, gel pieces were destained with 15 mM potassium ferrocyanide and 50 mM sodium thiosulfate. Protein reduction and alkylation were performed with 10 mM DTT at 56 °C for 45 min and with 55 mM IAA at room temperature for 30 min, respectively. Proteins were digested with trypsin (6.25 ng/mL) at 37 °C for 18 h. The peptide solution was acidified with FA and desalted by using C18-Stage-Tips columns. The samples were partially dried and stored at − 20 °C until analyzed by LC–MS/MS.

### Human tumoral cell lines and culture conditions

All the cell lines [Jurkat, T-cell leukemia (DSMZ ACC 282); Caco-2 (ATCC® HTB-37™)] were cultured at 37 °C in a humidified CO2 incubator (5% CO_2_) in complete RPMI media (Jurkat) or DMEM media (Caco-2) [(RPMI-1640 medium or DMEM supplemented with 10% (v/v) FBS and 1% (v/v) P-S]. When the cells reached 80% confluence, they were subcultured. For the adherent cells (Caco-2), 0.25% trypsin–EDTA was used to detach the cells. When necessary, the cells were counted using a Neubauer counting chamber and dyed with Trypan Blue. A modified culture medium was used for the Click it enrichment assays. DMEM without glutamine, methionine and cystine [supplemented with 10% (v/v) FBS 1% (v/v) P-S] and l-Azidohomoalanine was used when the cells were exposed to the NPs for 4 h to allow the protein labelling.

### Cell viability by MTT assay

To assess the cell viability after the incubation at different concentrations (0.1 and 1 mg/mL), an MTT assay was performed following standard protocols. Briefly, 2500 Caco-2 and 10 000 Jurkat cells per well were seeded in triplicate in 96-well plates (Caco-2 cells were seeded 24 h before the assay to allow attaching). Cells were incubated with the corresponding stimuli (NPs) for 24, 48 and 72 h. Once these periods of time elapsed, 20 μL per well MTT (5 mg/mL) were added in darkness. After 4 h MTT incubation, supernatant was removed and 200 μL per well DMSO were added to stop the reaction and the sample absorbance at 570 nm was determined by Gen5™ software (BioTek U.S., Winooski, VT, USA). The viability (percentage) for each concentration of nanoparticle was calculated with the following Eq. 1:

$$\frac{Abs. \,of \,cells\, with\, np-Abs. \,of \,cells\, without\, np}{Abs.\, of\, growth\, medium \,with \,np-Abs. \,of \,growth \,medium \,without \,np} \times 100$$

Equation 1: calculation of cell viability in MTT assays.

### Apoptosis assay and cell cycle by flow cytometry

To evaluate the viability by cytometry, 500,000 per well were seeded in 60 mm cell culture dishes, being exposed to the NPs after 24 h of incubation. The concentration of NPs and period of incubation used for this test was based on MTT assay’s results (0.1 mg/mL and 24 h). After the NP incubation period, the cells were collected (Jurkat) or detached (Caco-2) and centrifuging three times at 1200 rpm for 5 min. With each centrifugation the supernatant was discarded and replaced with PBS. In final centrifugation the PBS, sample was divided into two different tubes for both assays. For apoptosis assay, PBS was replaced with 200 μL of Annexin Binding Solution (ABS), incubating in the dark for 15 min. Afterwards, 5 μL of Annexin V FITC and PI were applied to each sample, incubating at room temperature in the dark for 15 min, and subsequently 200 μL more of ABS were added to each sample, to dilute the Annexin V FITC and/or IP. For cell cycle assay, PBS was replaced for 200 uL of Cycloscope™ Reagent and samples were incubated in dark for 15 min. The results were obtained by using a flow cytometer FACSCalibur and analyzed with InfinicytTM (Cytognos, Salamanca, Spain).

### Labeling for detection and identification of newly synthesized proteomes in the targeted cell lines.

To label cells, 200 000 cells (Jurkat and Caco-2) were cultured 12 wells’ plate with methionine free medium for 60 min at 37 °C to deplete methionine reserves. After this time, cells were exposed to azidohomoalanine (AHA) (35 μM) and the NPs (0.10 mg/mL) for 4 h. When incubation time was ended, cells were centrifugated (1200 rpm, 5 min) and the manufacturer’s instructions for Protein Enrichment Kit were followed. Resin functionalized with an alkyne derivative provided allowed the capture and the trypsinization of nascent proteins [46].

#### SDS-PAGE Separation for LC–MS/MS

A total of 20–25 μg of total protein (from hard or soft protein corona) were run on a 12% polyacrylamide precast Ready Gels (Mini-Protean TGX Precast Gels, Bio Rad Laboratories, Inc., USA) under reducing conditions for only 1 cm. Gels were stained in a 0.5% (w/v) Coomassie Bril-lant Blue solution. Polyacrylamide gels were then digitized with a gel reader and stored at 4 °C in an aqueous solution containing 1% (v/v) acetic acid until analysis.

### Detection of protein corona and detection and identification of newly proteomes with biorthogonal non-canonical amino-acid tagging azidohomoalanine (AHA)

Each gel lane was manually cutted and digested with sequencing grade trypsin (Promega) following the method described by Shevchenko et al., 1996, with slight modifications [19]. Briefly, Coomassie Blue gel plugs were detained with a working solution 1:1 (v/v) of 50 mM ammonium bicarbonate-acetonitrile. Next, dehydrated plugs with acetonitrile were treated with 10 mM dithiothreitol in 50 mM ammonium bicarbonate at 56 °C for 45 min and subsequently alkylated with 55 mM iodoacetamide in 50 mM ammonium bicarbonate at room temperature in the dark for 30 min. Proteins were digested with formic acid and desalted by using C18-stage tips columns [47]. The samples were partially dried and stored at − 20 °C until being analyzed by LC–MS/MS. Then, the trypsin digested proteins were analyzed by reversed-phase LC–MS/MS using an LTQ-Orbitrap MS/MS (Thermo Fisher Scientific, Waltham, MA). A nanoUPLC system (nanoAcquity, Waters Corp., Milford, MA, USA) coupled to an LTQ-Orbitrap Velos mass spectrometer (Thermo Fisher Scientific, San Jose, CA, USA) via a nanoelectrospray ion source (NanoSpray flex, Proxeon, Thermo) was used for reversed-phase LC–MS/MS analysis. Peptides were dissolved in 0.5% FA/ 3% ACN and loaded onto a trapping column (nanoACQUITY UPLC 2G-V/M Trap Symmetry 5 μm particle size, 180 μm × 120 μm C18 column, Waters Corp., Milford, MA, USA). Peptides were separated on a nanoACQUITY UPLC BEH 1.7 μm, 130 Å, 75 μm × 250 mm C18 column (Waters Corp., Milford, MA, USA) flow rate of 250 nL/min, gradient A formic acid 0.5% and B: ACN, from 1 to 40% B in 120 min. The LTQ-Orbitrap Velos was operated in the positive on mole applying a data-dependent automatic switch between survey MS scan and tandem mass spectra (MS/MS) acquisition. Survey scans were acquired in the mass range of m/z 400 to 1600 with 30,000 resolution at m/z 400 with lock mass option enabled for the 445.120025 ions [48]. The 20 most intense peaks having ≥ 2 change state and above the 500-intensity threshold were selected in the ion trap for fragmentation by collision-induced dissociation with 35% normalized energy, 10 ms activation time, q = 0.25, ± 2 m/z precursor isolation width and wideband activation. Maximum injection time was 1000 and 50 ms for survey and MS/MS scans, respectively AGC was 1 × 10^6^ for MS and 5 × 10^3^ for MS/MS scans. Dynamic exclusion was enabled for 90 s.

### LC–MS/MS data analysis

All raw files were converted to mgf using Proteowizard [49]. Data files were searched using Comet [50] via SearchGUI (v.3.2.10) [51] against the *Homo sapiens*; and PeptideShaker (v.1.16.2) [52] against a custom database combining the NeXtProt [53] database downloaded January 2020 with CrAP contaminant sequences (Uniprot_Rabbit_0009986_20180514; UniProt_Human_9606_20180202; UniProt_Bos_taurus_0009913_20180514).

Search parameters were set a follows carbamidomethylation of cysteines as fixed modifications, oxidation of methionine and acetylation of the protein N-terminal as variable ones, precursor and fragments mass tolerance were set to 10 ppm and 0.6 Da mass tolerances for precursor and product ions, respectively, and fully tryptic digestion with up to two missed cleavages. Regarding AHA labeling Up to 2 missed cleavages were allowed. Met oxidation (+ 15.9949) and N-terminal acetylation (+ 42.0106) were specified as variable modifications, and carbamidomethyl cysteine (+ 57.0125) as a fixed modification. In all Aha labelling experiments, AHA (− 4.9863) and L-2,4-diaminobutanoate (− 30.9768), a product of reduction of AHA, were specified as variable modifications for Met.

The mass spectrometry proteomics data have been deposited to the ProteomeXchange Consortium via the PRIDE partner repository [54] with the dataset identifier PXD026615 and 10.6019/PXD026615

### IONPs efficacy in animal model

Cisplatin derivatives and novel NPs synthesized in this report were tested “in vivo” in New Zealand rabbits in Animal Experimentation Support Service (SAEA) of the University of Zaragoza. Protocols for sample preparation, tumor induction, drug and tumor monitoring are detailed in supplementary file.

## Supplementary Information

**Additional file 1.**

## Data Availability

Additional file 1 is included with protocols used in the animal model assay, supplementary figures and supplementary tables. The full mass spectrometry proteomics data reported in this paper has been deposited to ProteomeXchange Consortium via the PRIDE [54] partner repository with the dataset identifier PXD026615.

## Abbreviations

AHA: Amino acid azidohomoalanine
BilPt: Platinum derivates conjugated with bile acids
CC: Cellular component
CisPtCl: [Pt(NH3)_2_Cl_2_]cisplatin compound
FBS: Fetal bovine serum
FEA: Functional enrichment analysis
GO-BP: Gen Ontology-Biological Process
IONPs: Iron oxide nanoparticles
IONP-Pt: IONPs conjugated with BilPt
IONP-Pt-Flu: IONPs with BilPt and fluorophore
NPs: Nanoparticles
SPECT: Single-photon emission computed tomography
