# Supplementary material for: Comprehensive and systematic characterization of multi-functionalized cisplatin nano-conjugate: from the chemistry and proteomic biocompatibility to the animal model
Source: J Nanobiotechnology. 2022 Jul 20;20:341. doi: 10.1186/s12951-022-01546-y (PMC9301860; doi:10.1186/s12951-022-01546-y)
Supplement: Supplementary file 1 — Additional file 1. [file 12951_2022_1546_MOESM1_ESM.docx]

# Comprehensive and systematic proteomic characterization of multi-functionalized cisplatin nano-conjugate: From the chemistry and biocompatibility to the animal model

# *Supplementary material*

# Supplementary protocols

## “In vivo” studies of IONPs efficacy

*Preparation of samples for in vivo administration*

All the stock solutions for in vivo injection contained 1.87wt% mannitol, 2.5wt% glucose and 0.88wt% NaCl, emulating solutions used in human therapy (Pharmacia Nostrum). The iron oxide content in NPs stock solutions for in vivo injections was 3088 mg(Fe_2_O_3_)/L (from ICP/OES analysis). The amount of Pt in stock solutions, as analyzed by ICP/OES was 351 mg(Pt)/L in CisPtCl ([PtCl_2_(NH_3_)_2_], Pharmacia Nostrum), 219 mg(Pt)/L in CisPtBil ([Pt(DCG)_2_(en)]), 515 mg(Pt)/L in NanoCisCl (IONP@BCP@[PtCl_2_(NH_3_)_2_]), and 266 mg(Pt)/L in NanoCisBil (IONP@BCP@[Pt(DCG)_2_(en)]).

*Animals*

New Zealand rabbits from the Animal Experimentation Support Service (SAEA) of the University of Zaragoza were used as animal models. All the experiments and procedures followed have been approved by the Ethical Committee for Experimental Animals of the University of Zaragoza (PI46/16). A total of twelve male rabbits, two months old, weighing about two kg were used. The animals were kept individually in approved cages, consuming standard feed and water *ad libitum*, with 12-hour light cycles and controlled environmental conditions of temperature (21-23 ºC) and relative humidity (55 %).

*VX2 tumor fragments*

Tumor fragments (1 x 1 mm, approximately) obtained at the Pharmacology unit of the Veterinary Faculty of the University of Zaragoza were used. For this, an initial tumor fragment, generously provided by Dr. David Melodelima (CERN, Unit 556, Lyon, France), was implanted in the liver of a carrier animal. The tumor mass developed (30 x 30 mm) was excised, separated from the surrounding healthy tissue and subsequently fragmented. These fragments were then preserved in RPMI 1640-glycerol (70:30) medium, at -150 ^o^C, thus ensuring genetic homogeneity.

*Tumor implantation*

Intrahepatic implantation of cryopreserved solid tumor fragments was carried out according a previously described technique [Parvinian, A.; Casadaban, L.C. and Gaba R.C. Development, growth, propagation, and angiographic utilization of the rabbit VX2 model of liver cancer: a pictorial primer and “how to” guide. Diagn. Interv. Radiol. 2014, 20(4), 335–340.  Doi: 10.5152/dir.2014.13415]. Briefly, animals were pre-anesthetized by intramuscular (IM) administration of 0.1 mg/kg of medetomidine (Medetor®), thus achieving good sedation and analgesia. After an induction period of 10 min, the animals were anesthetized by IM administration of a mixture of 25 mg/kg ketamine (Imalgene 100®) and 0.4 mg/kg butorphanol (Torphasol®). The surgical technique consisted of a sub-xiphoid incision of about 2 cm through the skin and muscles of the abdomen, through which the ventro-caudal end of the left hepatic lobe was visualized. A incision, 5 mm deep, was then made with the tip of an eye scalpel in the liver surface, and a single tumor fragment placed in the pocket created and gently pushed into the hepatic parenchyma with the tip of a fine forceps. Once the fragment was introduced, pressure hemostasis was performed for 30 seconds. Finally, muscular plane was closed by using continuous suture and skin with surgical staples.

*Drug administration*

Drug administration was performed on post-implantation day 10. Given the potential nephrotoxicity of CisPt and its derivatives, the classic clinical protocol for the administration of CisPt in patients undergoing chemotherapy was followed. Prior to the administration of the drug under study, an initial hydration period was performed by intravenous infusion (Intracath®) in the marginal vein of the ear of 0.9% NaCl supplemented with 15 mEq/L of KCl (SS+K) at a rate of 15 mL/kg/h for a period of 90 min. Immediately different drugs (CisPtCl, CisPtBil, NanoCisPtCl and NanoCisPtBil) or SS+K were administered at doses of 40 mg/m^2^ body surface area, dissolved in the necessary volume SS+K to allow administration in 1 h at a rate of 15 ml/kg/h. After this infusion, forced diuresis was performed by administration of mannitol (Mannitol 20®, Braun) at a dose of 1 mg/kg, diluted in SS+K at the same rate. Finally, the animal was kept under observation with an infusion of SS+K at the same rate until spontaneous elimination of urine was observed.

*Tumor monitoring*

Five ultrasound controls were performed on all the animals, at 10 days post-implantation, coinciding with the removal of staples, and subsequently at 11, 15, 17 and 18 days. This ultrasound monitoring made it possible to determine when the tumor began to be detected and to measure its size in order to quantify its growth over time. A Midray DP-10 ultrasound scanner equipped with a B-mode flat probe was used for these controls.

*Euthanasia, necropsy and sample collection*

Finally, after the last ultrasound control, the animals were euthanized by administration of an overdose (100 mg/kg) of sodium pentobarbital (Dolethal®, Vetoquinol), IV through the marginal vein of the ear. After the sacrifice of the animals, their necropsy was performed in order to take real measurements of the size of the masses, as well as to perform the exeresis of the tumor. Once removed, they were preserved in 10 % buffered formaldehyde (pH 7.4) and sent to the Anatomic Pathology Service of the Veterinary Faculty for their processing. The abdominal and thoracic cavity was also observed to evidence the presence of possible metastases and other relevant alterations. Once the tumors were separated from the main mass of the liver, it was observed that most of them had an ellipsoid shape, so their three main axes were measured, calculating their volume according to equation 2:

$$Tumor volume=\frac{4}{3} a b c$$

Equation 2: calculations for tumor volume estimation

Where a, b and c were the three axes measured. When the mass had a spheroid shape, only two axes were measured, and the volume was calculated with the major axis squared.

# Supplementary figures


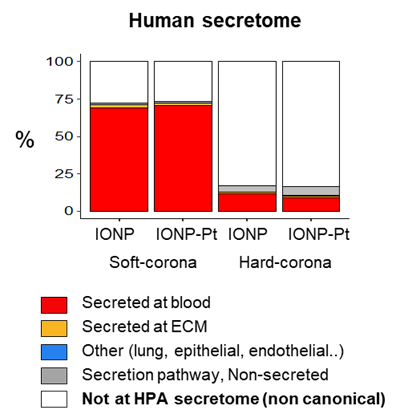


**Figure S1**: Bar plots summarizing human secretome analysis of protein corona of IONPs.


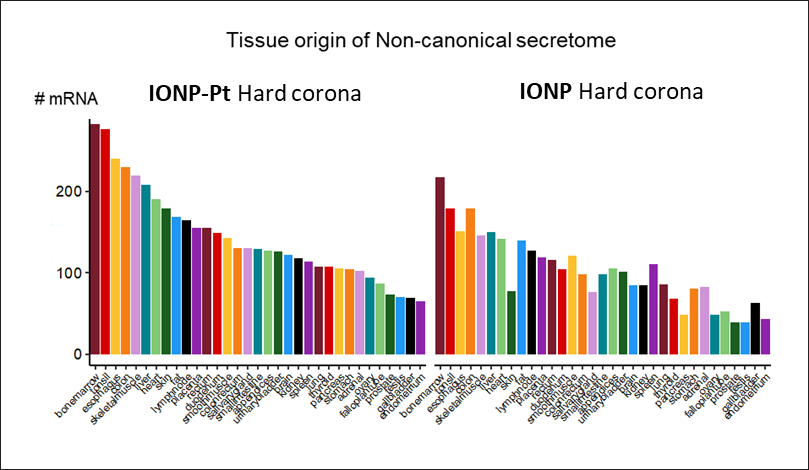


**Figure S2**: Bar plots summarizing the most frequent histological origin of hard corona non-secreted proteins. Bar plot Y-axis represents the total number of protein-coding transcripts primarily expressed at each tissue. Each transcript primary tissue expression is assigned by filtering highest 3^rd^ Quartile mRNA level along the 33 tissue-specific RNA-seq datasets available at HPA.


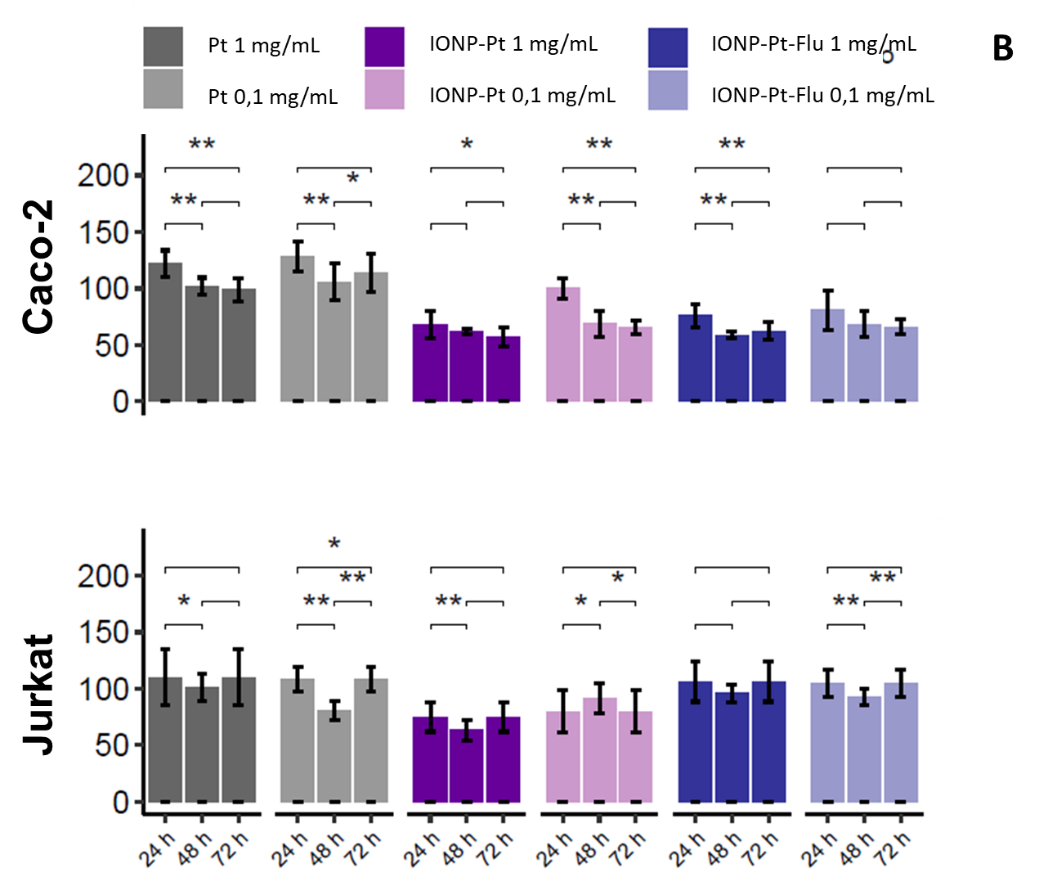


**Figure S3**. A) Viability assay performed in Caco-2 and Jurkat cancer cell lines using Pt precursor, IONP-Pt and IONP-Pt-Flu at different concentrations (1 and 0.1 mg/mL) incubating for 24-72 h. Results are reported as the mean number of live cells relative to the control (vehicle) from three independent experiments (significantly differences p * <0.05, ** <0.01)


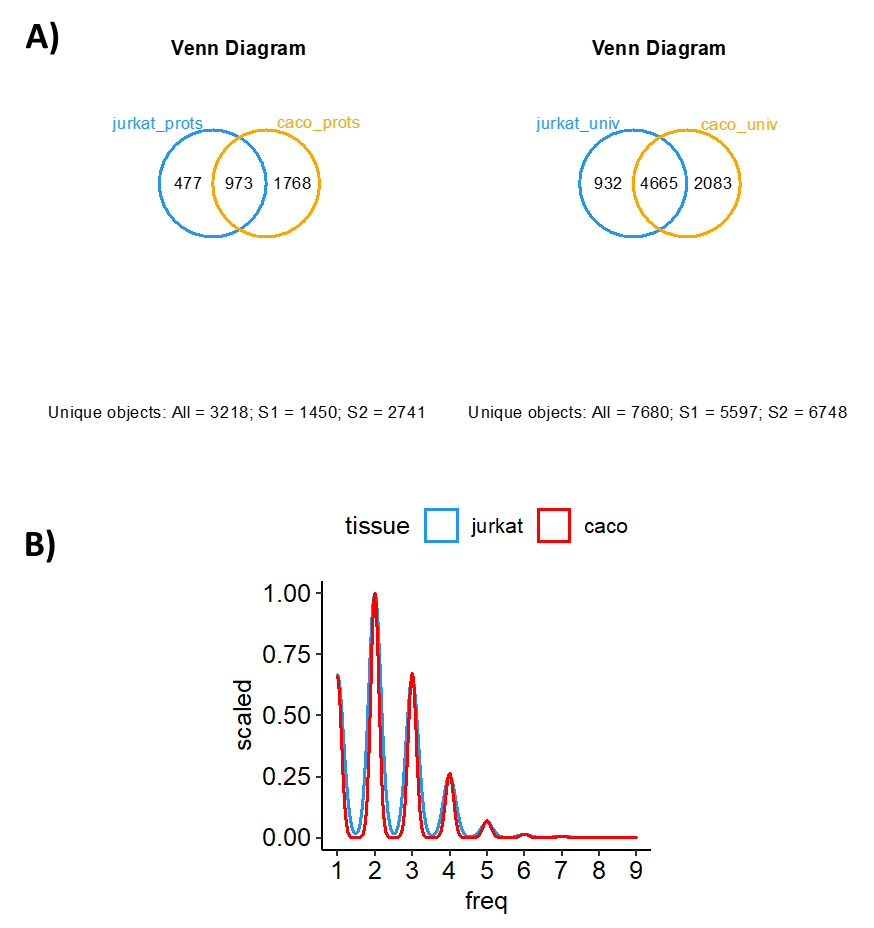


Figure S4: A) Venn diagrams with total number of proteins for each cell line. B)Comparison of density distributions of parent terms in GO repository of GO terms annotated in jurkat and caco cells. X axis represents the number parent terms of each annotation what indicates the hierarchical depth i.e., level of description detail of the functional annotation

# Supplementary tables

Table S1: Most representative functions derived from enrichment of FBS protein corona (hard-IONP *vs* soft-IONP-Pt)

| **GOterm** | **Frequency** | **Proteins** |
| --- | --- | --- |
| biological regulation | 25 | A2M/ACTB/ADIPOQ/AFM/AGT/C3/C9/CFH/CHIA/CLEC3B/CLU/CPB2/F2/F5/FGG/GSN/ITIH3/KLKB1/KNG1/LUM/ORM1/PLG/PROS1/SERPINA6/SERPINF1 |
| response to stress | 15 | ADIPOQ/C3/C9/CFH/CHIA/CLU/CPB2/F2/F5/FGG/KLKB1/KNG1/ORM1/PLG/PROS1 |
| regulation of biological quality | 15 | ADIPOQ/AFM/AGT/C3/CLU/CPB2/F2/F5/FGG/GSN/KLKB1/KNG1/PLG/PROS1/SERPINA6 |
| defense response | 9 | ADIPOQ/C3/C9/CFH/CHIA/F2/KLKB1/KNG1/ORM1 |
| proteolysis | 8 | A2M/C3/CLEC3B/CLU/F2/ITIH3/KLKB1/PLG |
| blood coagulation | 8 | CPB2/F2/F5/FGG/KLKB1/KNG1/PLG/PROS1 |
| hemostasis | 8 | CPB2/F2/F5/FGG/KLKB1/KNG1/PLG/PROS1 |
| response to wounding | 8 | CPB2/F2/F5/FGG/KLKB1/KNG1/PLG/PROS1 |
| wound healing | 8 | CPB2/F2/F5/FGG/KLKB1/KNG1/PLG/PROS1 |
| coagulation | 8 | CPB2/F2/F5/FGG/KLKB1/KNG1/PLG/PROS1 |
| regulation of body fluid levels | 8 | CPB2/F2/F5/FGG/KLKB1/KNG1/PLG/PROS1 |
| inflammatory response | 7 | ADIPOQ/C3/CHIA/F2/KLKB1/KNG1/ORM1 |
| regulation of response to stress | 7 | ADIPOQ/C3/CLU/F2/KLKB1/PLG/PROS1 |
| regulation of proteolysis | 6 | A2M/C3/CLEC3B/CLU/F2/ITIH3 |

Table S2: counting of detected proteins in each of the secretome conditions

Table S3: Proteins detected in AHA assay in signaling pathways related to cisplatin derivates.

| Condition | ID | Description | GeneID |
| --- | --- | --- | --- |
| Jurkat IONP-Pt | GO:0006303 | double-strand break repair via nonhomologous end joining | H4C1/H4C2/H4C3/H4C4/H4C5/H4C6/H4C8/H4C9/H4C11/H4C12/H4C13/H4-16/H4C14/H4C15/PRKDC |
|  | GO:0006986 | response to unfolded protein | EIF2S1/HSP90AA1/HSP90AB1/ASNS/HSPD1/HSPA5/HSPA8/HSP90B1/HSPA9/VCP |
|  | GO:0018149 | peptide cross-linking | ANXA1/KRT1/KRT10/DSP/KRT2 |
|  | GO:0006984 | ER-nucleus signaling pathway | EIF2S1/ASNS/HSPA5/HSP90B1 |
| Jurkat IONP-Pt-Flu | GO:0009165 | nucleotide biosynthetic process | ACOT7/PFAS/COX2/PNP/HPRT1/ADA/ALDOA/TYMS/ATP5F1B/PARP1/PDHB/MTHFD1/IMPDH2/PKM/NME1/CTPS1/GART/PAICS/RRM1/ACAT1/ATP5F1A/CMPK1/ATIC/DCTD/SHMT1/ATP5F1C/GARS1/IDH2/GMPS/ACLY/VCP/PPAT/KARS1/GUK1/STOML2 |
|  | GO:0033260 | nuclear DNA replication | PCNA/MCM3/RPA1/MCM4/MCM5/MCM7/RPA3/FEN1/RFC3/MCM2/MCM6/UPF1/RTF2 |
|  | GO:0072593 | reactive oxygen species metabolic process | COX2/CAT/GNAI2/HSP90AA1/HSP90AB1/GNAI3/GSTP1/G6PD/POR/VDAC1/PRDX6/PRDX5/PRDX3/PRDX2/DNM2/HDAC4/GRB2/RAC1/HBA2/HBA1/PRDX1/PARK7 |
| Caco-2 IONP-Pt | GO:0009150 | purine ribonucleotide metabolic process | PKM/ATP5F1B/GAPDH/ENO1/VCP/PGK1/ATP5F1A/ACLY/TPI1/PFKP/HMGCS1/ATIC/GPI/ACAT1/LDHA/PARP1/PDHB/HSD17B12/ALDOA/STOML2/NUP93/NME1/GNAI3/ATP5F1C/MCCC2/ACSL3/ATP5PO/HPRT1/TPR/RHOA/HMGCS2/SUCLG2/NUP37/ATP5F1D/GART/EIF6/SUCLG1/PGM1/ACSL5/NUP205/GMPS/PGAM1/TJP2/ADSS2/CYC1/EPHA2/DBI/NUP188/NUP133/NUP98/OGT/SLC25A13/PFKFB2/NUP107/DLAT/DLD/RANBP2/FAR1/PFKL/GALK1/APRT/STAT3/PDHA1/SEC13/SLC25A1/HINT1/ADK/PPAT |
|  | GO:0006260 | DNA replication | DHX9/RRM2/MCM2/MCM7/MCM3/PPP2R1A/MCM6/SUPT16H/RRM1/PCNA/STOML2/SMC3/GTPBP4/UPF1/RBM14/SSRP1/MCM4/TOP1/PDS5A/RPA1/FEN1/MCMBP/RTF2/GINS2/S100A11/CACYBP/NASP/RBBP4/CDK9/RECQL/NUP98/DNAJC2/BOD1L1/CDK2/RAC1/POLD1/KAT7 |
|  | GO:0072321 | chaperone-mediated protein transport | HSPA8/CLU/BAG3/TIMM13 |
| Caco IONP-Pt-Flu | GO:0016126 | sterol biosynthetic process | CYB5R3/APOA1/APOE/G6PD/POR/CES1/FDFT1/FASN/ACLY/HMGCS2/CNBP/RAN/HMGCS1/ACACA/IDI1/KPNB1/CYP51A1/ACAT2/DHCR7 |
|  | GO:0097006 | regulation of plasma lipoprotein particle levels | AP2A2/ACSL3/AP2A1/AGT/LDLR/APOA1/APOE/P4HB/ANXA2/CSK/PLTP/HNRNPK/ARF1/CLTC/EHD1 |

1. Diez P, Gonzalez-Munoz M, Gonzalez-Gonzalez M, Degano RM, Jara-Acevedo R, Sanchez-Paradinas S, et al. Functional insights into the cellular response triggered by a bile-acid platinum compound conjugated to biocompatible ferric nanoparticles using quantitative proteomic approaches. Nanoscale. 2017;9(28):9960-72.
